# Nuclear Mechanotransduction in Cancer Radiation Response: Linking Nuclear Envelope Dynamics to DNA Damage Repair

**DOI:** 10.3390/ijms27219780

**Published:** 2026-10-30

**Authors:** Feiyan Xu, Yang Xiang, Wentian Huang, Ping Hu, Shun Wang, Kun Cao, Zhijie Wan, Yanyong Yang, Fu Gao, Yuanyuan Chen

**Affiliations:** 1 Department of Radiation Medicine, Naval Medical University, Shanghai 200433, China; 18166998570@163.com (F.X.); hwtian2001@163.com (W.H.); hh2062608@163.com (P.H.); wsqtql12@163.com (S.W.); seeu_tomorrow@163.com (K.C.); wanzhijie@smmu.edu.cn (Z.W.); yyyang2010@163.com (Y.Y.); 2 Department of Burns, Changhai Hospital, Naval Medical University, Shanghai 200433, China; 15721570511@163.com; 3 School of Public Health, China Medical University, Shenyang 110122, China

**Keywords:** nuclear mechanotransduction, radiation response, nuclear envelope, nuclear lamina, DNA damage repair, chromatin, tumor microenvironment

## Abstract

Tumor mechanics influence radiation response, yet no single mechanical parameter predicts radiosensitivity, and nominally identical variables report opposite results. The gap is not a shortage of mechanisms but the absence of a validated link between a measurable nuclear state, the fidelity of damage processing, and long-term reproductive outcome. This review follows mechanical signals from adhesion and the cytoskeletal systems through the linker of nucleoskeleton and cytoskeleton (LINC) complex, nuclear pores and lamina to chromatin, the nucleolus and DNA repair, asking at each step whether the mechanical input could change how radiation damage is processed rather than how much is produced. Mechanical manipulation combined with irradiation in the same system remains uncommon; most evidence establishes mechanosensitive components without connecting them to a radiation outcome, and this asymmetry accounts for much of the apparent conflict in the literature. We therefore sort the evidence into three levels of support, propose a three-state framework of nuclear mechanical homeostasis (insufficient structural support, maladaptive structural constraint, coordinated integrity and plasticity), and specify the experiments that would falsify it. Nuclear mechanotransduction is better described as a context-dependent adaptive network than as a single determinant of radiosensitivity, and matrix-normalizing, Src-directed and ultrasound-based interventions remain unvalidated for radiotherapy.

## 1. Introduction

### 1.1. Radiotherapy Resistance and Established Determinants

A global population-based analysis integrating GLOBOCAN 2022 incidence data with radiotherapy-utilization models estimated approximately 20 million new cancer diagnoses in 2022 and, assuming a 50% utilization rate, a need for radiotherapy in about 10 million patients [1]. Radiotherapy outcomes nevertheless remain shaped by treatment technology, intrinsic tumor biology, and the tumor microenvironment [2,3]. Local failure may reflect pre-existing low-sensitivity subpopulations, irradiation-induced microenvironmental change, clonal selection under treatment pressure, or adaptive evolution [3,4]. Because these processes overlap in time, recurrence alone cannot distinguish selection of a pre-existing low-sensitivity population from adaptation acquired during treatment—a distinction that matters when a mechanical variable is proposed as a stratifying feature.

Radiation response depends on absorbed dose, radiation quality, oxygenation, cell-cycle state, lesion complexity, and the DNA damage response (DDR) [5,6,7,8]. Genetic alterations in DNA repair, checkpoint control, and cell death pathways also contribute to treatment sensitivity. In addition, DNA methylation, histone modifications, chromatin remodeling, and metabolic adaptation can alter how tumor cells process radiation-induced damage [2,3,4,5,6,7,8]. In vivo, radiation-killed tumor cells expose or release damage-associated molecular patterns (DAMPs) such as calreticulin, high-mobility group box 1 (HMGB1), and extracellular ATP, and the resulting immunogenic cell death (ICD) can convert a sublethal dose into an immune-relevant event whose outcome depends on host sensing and on how efficiently the released DNA is cleared [9,10]. These determinants do not act independently. For example, the relative contribution of homologous recombination (HR) and non-homologous end joining (NHEJ) depends on cell-cycle state, lesion type, chromatin context, and pathway availability [6]. The same caveat applies to immunogenic cell death, which is detectable in immunocompetent hosts but largely invisible to clonogenic assays performed in culture. This context dependence cautions against interpreting any single repair marker as a complete measure of radiosensitivity.

Experimental studies link mechanical conditions to radiation-associated DNA damage responses or survival [11,12,13]. However, the independent contribution of mechanics to radiosensitivity remains incompletely resolved. Tumor physical conditions can vary spatially and with disease state [14,15,16], and stromal interventions can alter solid stress, interstitial pressure, and perfusion in experimental tumor models [17,18]. Mechanical heterogeneity persists after such interventions, and its persistence is itself a variable worth measuring rather than assuming. Tumor physical conditions therefore represent a potentially relevant, but insufficiently resolved, source of radiation-response heterogeneity.

### 1.2. Why Tumor Mechanics May Influence Radiation Response

Spatial heterogeneity in tissue stiffness has been demonstrated in mammary tumor models and human breast tissues [14,15]. A single bulk stiffness value cannot fully describe the tumor physical environment. Matrix elasticity [16,17,18,19], viscoelastic behavior in engineered tumor matrices [20], and solid stress [17,18,19,20,21] are distinct mechanical features; interstitial pressure and extracellular matrix (ECM)-dependent transport further influence the local physical environment [18,22]. The direction of these effects is not always intuitive: in the same tumor, a stiff region may be poorly perfused while a soft region carries high interstitial pressure, so the two signals that a cell integrates can point in opposite directions. Mechanical heterogeneity is therefore not noise around a mean stiffness but a set of partly dissociable variables, and it may influence how cells sense and respond to their surroundings.

When these conditions are manipulated experimentally alongside irradiation, the reported direction of the effect varies across models [11,12,20,23,24]. Greater matrix stiffness has been associated with reduced radiation responses in some experimental systems [11,12,23], whereas other studies report resistance to irradiation or greater post-irradiation survival in softer environments [20,24]. Across these studies, differences in tumor type, cellular state, matrix formulation, culture dimensionality, irradiation conditions, and outcome measures limit direct comparison [11,12,20,23,24]. Our own reading of this literature is that the discordance is rarely a genuine contradiction; it usually tracks differences in which nuclear property the assay happened to report, a point we return to in Section 6. The available findings therefore do not support a universal relationship between stiffness and radiosensitivity.

Direct experiments support cytoskeleton-to-nucleus force transmission [25,26], force-dependent nucleocytoplasmic transport [27,28], mechanical support by the nuclear lamina [29,30], and nuclear-envelope rupture during confined migration [31,32]. Constricted migration has also been associated with repair-factor depletion and DNA damage [33]. These interfaces provide plausible routes through which physical conditions could influence nuclear damage processing. However, individual interfaces have been established under different cellular, loading, and damage conditions; their combined effects on radiation response require validation in the relevant tumor system. In particular, it remains unclear whether nuclear mechanical state changes radiation response mainly by altering damage formation, by changing the recruitment and use of repair pathways, by affecting repair fidelity, or by influencing post-damage recovery and cell fate.

These interfaces do not specify which mechanical input drives them, and matrix stiffness, although the most frequently measured, is not the only mechanical input experienced by tumor cells in vivo. Blood flowing through the disorganized intratumoral microvasculature imposes hemodynamic shear stress on endothelial cells, and interstitial flow driven by elevated interstitial pressure imposes lower-magnitude shear along perivascular and lymphatic routes [34,35]. Both are wall stresses rather than matrix properties, and both fluctuate with perfusion, vasomotion, and vessel collapse, conditions that also determine oxygen delivery and drug access. Shear forces of this kind are transmitted through the glycocalyx, integrins, and PIEZO1-dependent pathways that converge on Src and Yes-associated protein (YAP) [36], and they can remodel the nucleus itself: circulating tumor cells surviving fluid shear show nuclear expansion and altered deformability, and this adaptation tracks with survival rather than with matrix stiffness [37]. Shear also changes radiation response directly. Laminar flow applied to tumor cells before irradiation increases radiosensitivity, and the effect depends on integrin and focal adhesion kinase (FAK) signaling rather than on oxygenation alone [38]. That evidence comes from flow-chamber and microfluidic systems rather than from irradiated tumors in vivo, but it establishes that shear is a radiation-relevant variable and not only a confounder for perfusion. Because shear stress changes with the same vascular normalization that alters oxygenation, mechanical studies that ignore flow risk attributing a shear effect to matrix mechanics. Section 2.2 examines shear alongside matrix mechanics.

### 1.3. From Nucleomechanical Perturbation to Malignant Transformation

If nuclear mechanics shapes the response of an established tumor to irradiation, the same properties may act considerably earlier, when a normal or premalignant cell first loses nuclear constraint. The strongest available evidence concerns nuclear-envelope structural defects. In ovarian surface epithelial cells, a defect in nuclear-envelope organization precedes and appears to drive the aneuploidy that characterizes early carcinogenesis, and the defect is detectable before overt malignant transformation [39]. Lamina composition is part of this picture: A-type lamin deficiency destabilizes responses dependent on p53-binding protein 1 (53BP1) and ataxia-telangiectasia mutated (ATM) and thereby permits damage to persist through mitosis [40,41,42], while excess lamin B1 sequesters 53BP1 away from lesions [43]. Either direction of imbalance can favor the propagation of unrepaired or misrejoined chromosomes.

Chromatin contributes a second, complementary layer. Heterochromatin remodeling can provide mechanoprotection: stretch-induced loss of heterochromatin marked by histone H3 lysine 9 trimethylation (H3K9me3) softens the nucleus, whereas blocking this response increases DNA damage [44]. This observation reframes chromatin not only as a determinant of repair accessibility (Section 4.2) but as a structural element whose adaptive reorganization can protect against mechanically induced genome instability. Sustained mechanical signaling supplies a third layer. YAP and TAZ (transcriptional coactivator with PDZ-binding motif) activation, actomyosin tension, and matrix remodeling form a feed-forward loop that stabilizes a transformed state once it is established [11,45,46], and confined migration through narrow spaces generates both DNA damage and repair-factor depletion that can be inherited by daughter cells [31,32,33]. Importantly, most of this evidence is correlative in human tissue and mechanistic only in engineered or immortalized models; whether nucleomechanical perturbation initiates transformation in a primary human cell, rather than accompanying it, remains an open question that the assays proposed in Section 7 could address.

### 1.4. Scope and Organization of Evidence

This narrative review traces the relationships among tumor mechanics, cellular mechanosensing, cytoskeletal force transmission across all three filament systems (actin, intermediate filaments, and microtubules), nuclear structure, chromatin state, the DNA damage response, and post-irradiation cell fate. Both sensitization and protection are considered, including findings that point in opposite directions. Rather than cataloging studies by model system, we distinguish three levels of support and invoke them explicitly wherever the level changes how a result should be read. **Level I** evidence combines controlled mechanical manipulation and irradiation with a damage-processing endpoint in the same system. **Level II** evidence shows a mechanosensitive nuclear or damage-response mechanism, or links a mechanical variable to a radiation outcome, without measuring a matched damage-processing endpoint in the same system. **Level III** comprises integrated hypotheses that connect findings across models and remain untested as such. The levels classify claims; the categories used later to describe study designs classify the studies themselves. The two are related but not interchangeable, and the difference is set out in Section 6.6. The levels are applied at those points in Section 4, Section 6 and Section 7, where the distinction determines the conclusion. We do not label every study, because many designs fall between levels and a label would imply a precision that the underlying experiments do not have.

The central knowledge gap is a missing validated link between measurable nuclear mechanical states, repair fidelity, and long-term survival after irradiation. The review asks whether nuclear mechanotransduction can alter radiation response through nuclear-envelope integrity, chromatin organization, repair-factor recruitment, damage-site mobility, and post-damage recovery. These questions are organized around a three-state model of nuclear mechanical homeostasis, introduced in Section 6 and given operational definitions in Section 6.6. The model assumes no fixed thresholds, no universal relationship between stiffness and radiosensitivity, and no predetermined ranking of nuclear states. It is a framework for evaluating evidence and generating testable comparisons.

The translational value of this framework depends on defined nuclear mechanical phenotypes improving the prediction of tumor control or identifying interventions that sensitize tumors without increasing normal-tissue injury (Figure 1). Both outcomes remain to be demonstrated. The main question is this: under which cellular, mechanical, and radiation conditions does nuclear mechanotransduction alter the quality of DNA damage processing and the long-term fate of irradiated cells? The following sections trace the pathway from tumor mechanics to nuclear structure, DDR, innate immune signaling, and cell fate, and they deliberately keep direct radiation evidence separate from adjacent mechanistic evidence and from hypotheses that remain untested.

## 2. Evidence Linking Tumor Mechanics to Radiation Response

The matrix does not influence radiation response through stiffness alone. Its composition, architecture, viscoelastic behavior, solid stress, and transport properties affect how cells adhere to the matrix, generate force, and transmit that force toward the nucleus. Fluid movement through the same tissue adds a wall stress that is not captured by any matrix measurement. This section follows these connected levels of regulation, from tissue-scale mechanics and intratumoral fluid shear to cell-surface signaling, actomyosin contractility, ion-channel activation, and transcriptional adaptation, and then to the intermediate-filament and microtubular systems that anchor and position the nucleus.

### 2.1. Extracellular Matrix Mechanics and Mechanical Heterogeneity

Collagen crosslinking and fiber organization alter integrin adhesion and resistance to deformation, while growth-induced solid stress compresses vessels and impairs perfusion [16,17,19,21]. Hyaluronan generates swelling under confinement and also exerts receptor-mediated effects: in pancreatic cancer models, its enzymatic depletion reduced interstitial pressure and improved vascular function and drug delivery [18], and in esophageal squamous carcinoma cells, hyaluronan and processes associated with CD44 and the receptor for hyaluronan-mediated motility (RHAMM) were linked to focal adhesions, filopodia and proliferation [47]. Tissue-scale effects therefore involve pressure and transport rather than matrix softening alone, and matrix assembly itself influences interstitial transport [22]. The same ambiguity applies to collagen: increasing its concentration modifies ligand presentation, fiber architecture and diffusion at once, so a nominal modulus cannot be treated as the independent variable when the intervention also changes composition, ligand availability or transport.

Three-dimensional models illustrate these interactions. In a bioprinted breast cancer model, cancer-associated fibroblasts promoted matrix deposition, stiffening and radioprotective paracrine effects [48]; in a viscoelastic glioblastoma (GBM) model, radioresistance in soft matrices was associated with chromatin condensation, whereas chemotherapy responses followed a different pattern in stiffer environments [20]; and in SiHa cells cultured on 0.5, 5 and 25 kPa substrates, morphology, proliferation and radiation response changed concurrently [49]. These findings establish the importance of material context, but differences in treatment, culture dimensionality and endpoint preclude a single stiffness-response curve. Biomimetic GBM hydrogels varying material conditions, oxygenation, hyaluronan fragments and neighboring neural cells showed greater irradiation-associated damage signals in soft matrices and context-dependent metabolic responses [50]; such measurements reveal damage and stress states but do not substitute for colony formation, and spatial studies must register material properties against oxygenation, proliferation and cellular composition before local mechanics can be credited with independent information.

Opposing findings also have mechanistic support. Drain et al. linked resistance in soft environments to reduced proapoptotic c-Jun N-terminal kinase (JNK) activity and increased nuclear factor κB (NF-κB) activity [24]; the study combined matrix manipulation, organoids and animal experiments, but transplantation with NF-κB inhibition is not a test of stiffness alone, so the results connect soft environments with survival signaling without showing that changing stiffness independently improves response. Hepatocellular carcinoma (HCC) studies point the other way: in three-dimensional Huh7 collagen cultures, greater stiffness was associated with a weaker irradiation response, and lysyl oxidase inhibition with sonoporation altered both material properties and post-irradiation death [23], although collagen formulation, enzyme biology and ultrasound exposure changed together. Wang et al. linked greater stiffness to PFKFB3-related damage processing in 6, 10 and 16 kPa hydrogels [12], and Deng et al. identified suppression of damage-site ubiquitin signaling at low stiffness [13]; the repair mechanisms involved are considered in Section 4 rather than inferred from stiffness alone.

In a CNE-1 collagen-alginate three-dimensional comparison, Fang et al. reported higher post-irradiation surviving fractions in the softer formulation [51]. Material composition and baseline plating efficiency also differed, and the title of the corresponding figure conflicts with the direction reported in that paper’s Results, so confounding and inconsistent reporting limit attribution to stiffness and mechanistic generalization. Matrix-associated responses can reflect different combinations of load bearing, biochemical signaling, metabolism and transport; independently varying elasticity, viscoelastic relaxation, ligand density, pore structure and oxygenation, with unirradiated controls, cell-cycle measurements and long-term survival endpoints, can separate them. Anatomical labels such as tumor core, margin or invasive front do not define uniform stiffness or sensitivity, and regional differences require direct measurement rather than anatomical inference.

### 2.2. Hemodynamic Shear Stress and Interstitial Flow as Distinct Mechanical Inputs

Tumor blood flow is not a background variable. The disorganized, leaky, and intermittently collapsing microvasculature of solid tumors produces wall shear stresses that vary over orders of magnitude between vessels, and between one moment and the next within the same vessel [35]. Interstitial flow, driven by elevated interstitial pressure and by osmotic gradients across the capillary wall, adds a lower-magnitude but continuous shear along perivascular, perineural, and lymphatic routes. Both are transmitted at the cell surface rather than through the matrix: the glycocalyx and integrins transduce the tangential load, PIEZO1 channels open in response, and downstream signaling converges on Src, RhoA, and YAP [34,36]. In three-dimensional culture, fluid stresses are sufficient to redirect migration direction and to set the mode of motility independently of matrix stiffness [34].

Shear stress also reaches the nucleus. Shear stress lowers the survival of suspended tumor cells, while histone acetylation-mediated nuclear expansion limits shear-induced apoptosis [37]. This is one of the few contexts in which a mechanical input, a nuclear structural adaptation, and a survival outcome have been measured in the same cells. Two consequences follow for radiotherapy. Shear-driven Src and YAP activity overlaps directly with the adhesion signaling implicated in repair (Section 2.3), so any intervention that alters perfusion, whether vascular normalization, antiangiogenic therapy, or stromal depletion, changes oxygen delivery and this signaling axis at the same time. Intratumoral flow is also fluctuating and directionally complex, which makes a static shear value as uninformative as a single bulk stiffness. We are not aware of a study that has combined controlled shear, nuclear phenotyping and clonogenic survival after irradiation in the same tumor model; this is a specific gap rather than a marginal one, and Section 7.2 proposes how to close it.

The next mechanistic step is to determine how these tissue-scale conditions are converted into intracellular force. Integrins and focal adhesions provide one such interface by coupling ECM composition and deformation to the actin cytoskeleton.

### 2.3. Integrin and Focal-Adhesion Signaling

Integrins connect extracellular adhesion to actin, with talin and vinculin forming important load-bearing elements. Single-molecule stretching shows that talin unfolding exposes vinculin binding sites, while tension sensors reveal molecular loads carried by adhesion proteins [52,53,54,55]. The mechanical chain is now resolved in some detail: talin’s rod domain unfolds under tension to expose cryptic vinculin-binding sites, and force is required for the interaction rather than merely permitted by it [56]; vinculin recruitment in turn reinforces talin engagement with the actomyosin machinery, so that adhesion maturation is a mechanically self-reinforcing process [57]. α-Actinin contributes a parallel route, crosslinking actin and transmitting force to integrins, and its activation likewise depends on force-induced conformational change [58,59]. FAK/Src participate in adhesion-related kinase signaling and complex organization; FAK signaling can also be manipulated optogenetically [60]. FAK phosphorylation, adhesion size, and force across adhesions are consequently related but distinct measurements. This distinction matters for interpretation: an increase in phospho-FAK reports kinase activity, whereas an increase in talin or α-actinin tension reports load, and only the latter is a mechanical measurement in the strict sense.

Several models support radiation-related roles for β1-integrin. Its blockade enhanced irradiation effects in three-dimensional breast cancer cultures and xenografts [61], while another study linked NF-κB to β1-integrin-associated radiation survival [62]. In head and neck squamous carcinoma, β1-integrin inhibition altered FAK–cortactin signaling and produced clonogenic radiosensitization [63]. Breast epithelial models further connected β1-integrin to RAD51 availability and HR-related phenotypes through RING1-associated protein degradation [64]. These results connect adhesion signaling with DDR and survival, but they do not identify the mechanical load transmitted from the adhesion to the nucleus. Receptor blockade, kinase inhibition, and substrate softening are frequently treated as interchangeable “anti-adhesion” interventions; in practice each removes a different part of the chain.

FAK effects depend on genotype and cellular compartment. FAK loss or inhibition in KRAS-mutant lung cancer was associated with persistent damage and radiosensitization [65], whereas FAK loss in a particular advanced squamous carcinoma model enhanced p53–p21 responses and accompanied resistance [66]. In pancreatic cancer models, FAK inhibition also improved radiation-related responses through stromal and immune remodeling [67]. Discriminating tumor-cell signaling, adhesion load bearing, and host effects requires target- and compartment-specific perturbations rather than attribution based only on total FAK activity. Focal adhesions transmit force through actin, but the magnitude and direction of that force depend on cytoskeletal contractility; the next section therefore considers the RhoA–ROCK (Rho-associated kinase) system and its connection to the perinuclear actin cytoskeleton.

### 2.4. RhoA–ROCK-Dependent Actomyosin Contractility

RhoA–ROCK signaling promotes actomyosin contractility through regulation of myosin light chain phosphorylation and myosin phosphatase [68,69]. The perinuclear actin cap and its connection to the linker of nucleoskeleton and cytoskeleton (LINC) complex couple cytoskeletal organization to nuclear shape: adhesion micropatterning, contractility inhibition, and LINC perturbation demonstrate its contribution to shape maintenance [26,70]. Sustained material conditions are also associated with lamin A abundance and nuclear mechanical adaptation [71]. These observations define a route from extracellular material conditions to nuclear deformation, but they do not by themselves establish an effect on post-irradiation repair or survival—a gap that recurs throughout this section.

Osaki et al. compared HeLa cells stably expressing inhibitory or activating RhoA mutants and found reduced post-irradiation clonogenic survival under both persistent perturbations [72]. This suggests that deviations in RhoA activity can disrupt a state supporting radiation survival in that model; it does not demonstrate a universal optimal tension range. Other studies connect RhoC–ROCK2 to DDR and radioresistance in cervical cancer [73], and Syx–RhoA–Dia1 signaling to cell cycle, YAP/TAZ, DNA damage, and treatment response in GBM [74]. The bidirectionality of the Osaki result is worth dwelling on: both too little and too much RhoA activity were deleterious, which is more consistent with a homeostatic window than with a monotonic dose–response, and this is one of the observations that motivates the framework in Section 6.

In normal human fibroblasts, fasudil was radioprotective and altered HR-related measurements and RAD51/BRCA1 responses [75]. This drug-specific finding is not interchangeable with evidence concerning the tumor RhoC–ROCK2 pathway. Rho/ROCK affects contraction, proliferation, and migration, so survival changes may involve parallel processes. Molecular tension, nuclear deformation, target perturbation, and long-term colony formation measured together are needed before the contribution of nuclear mechanics can be separated from these parallel effects.

### 2.5. Mechanosensitive Ion Channels and Ca^2+^ Signaling

Contractile force is one route by which mechanical information enters the cell; ion channels open another, and they respond within seconds rather than minutes. PIEZO1/2 are major components of mechanically activated cation channels [76], and TRPV4 participates in endothelial reorientation under cyclic stretch [77]. Calcium signaling also regulates adhesion organization: calcium/calmodulin-dependent protein kinase II (CaMKII) perturbation affects focal adhesion turnover in fibroblasts [78], while proline-rich tyrosine kinase 2 (PYK2) responds to calcium through calmodulin-associated binding and self-association [79]. These studies establish adjacent mechanisms connecting membrane mechanosensing with calcium-dependent effectors, yet none of them establishes a post-irradiation resistance pathway in cancer, and the gap between membrane channel activity and nuclear outcome remains a matter of inference.

Yagi et al. combined hypertonicity, PIEZO1 knockdown, and pharmacological interventions in B16 melanoma, measuring DDR and colony formation after X-rays; Yoda1 combined with irradiation also suppressed tumor growth in mice [80]. The study therefore includes genetic evidence and a long-term reproductive endpoint beyond short-term pharmacological phenotypes. Nevertheless, cells returned to isotonic conditions before irradiation; hypertonicity is not a purely mechanical input, and GsMTx4 is not PIEZO1-specific. The mouse regimen used three 1 Gy whole-body exposures and does not directly represent clinical local radiotherapy. In the HCC study of Wang et al., ITGB1/PIEZO1 perturbation affected PFKFB3 expression and glycolysis-related measurements [12], providing another connection between mechanosensing and metabolism. Channel contributions to radiation endpoints and specificity for mechanical input should therefore be evaluated separately, and complete mediation between PIEZO1, CaMK/PYK2, and nuclear DDR remains to be tested. Calcium-dependent signaling can modify adhesion and metabolism over minutes, whereas YAP/TAZ integrates mechanical inputs over hours through transcription—the next level of the pathway from force sensing to cellular adaptation.

### 2.6. YAP/TAZ-Mediated Cellular Adaptation

Mechanical inputs also reach transcription. The two paralogs, YAP and TAZ, are the principal mechanically regulated transcriptional effectors. Matrix stiffness, cell spreading, and actin organization influence nuclear localization and TEA domain transcription factor (TEAD)-associated transcription; direct nuclear force can also promote YAP entry through nuclear pore transport [27,45]. Mechanical history can leave persistent phenotypes: stem cell experiments show that stimulus duration and previous environments influence subsequent fate [46]. Persistence of this kind is a testable variable in radiation biology rather than an established mechanism, and it cannot be equated directly with tumor radioresistance.

In glioma stem cells, Zhang et al. combined material manipulation with YAP1/TEAD1, IQGAP3, and SOX2 experiments to connect matrix stiffness with stemness and post-irradiation damage/growth phenotypes [11]. This is a mechanistic advance within the same tumor context rather than a synthesis of unrelated findings. It does not, however, establish a necessary nuclear lamina intermediate or the fidelity of HR or classical non-homologous end joining (c-NHEJ), and the distance between a growth phenotype and a repair-fidelity phenotype remains unbridged.

Other radiation studies show that YAP–FGF2 signaling can support glioma damage processing and resistance in vivo [81], and that YAP1 inhibition alters DDR and survival in triple-negative breast cancer [82]. Non-small-cell lung cancer (NSCLC) studies implicate ITGB1-related YAP1 programs and irradiation-induced YAP/TEAD4–NRP1 transcription [83,84]. YAP/TAZ can also affect RAD51 expression and HR through NRP2-related signaling, while TAZ has been associated with NHEJ and radioresistance in esophageal cancer [85,86]. These outputs encompass transcriptional and survival adaptation, although the extent of mechanical manipulation varies among studies—in several, the “mechanical” arm is a substrate change that also alters adhesion density and proliferation.

Acute nuclear translocation, sustained transcriptional adaptation, and mechanical memory have different temporal definitions. Mechanical memory requires persistence after removal of the original environment [46]; a single post-irradiation YAP localization measurement cannot distinguish an irradiation-induced response from a pre-existing mechanical state. Pretreatment histories followed by a common final environment provide a straightforward test of whether persistence changes the subsequent radiation response.

YAP can also produce opposing repair outputs. Shu et al. showed that Ku70 competes with TEAD4 for YAP binding, while YAP can promote SMURF2-dependent poly(ADP-ribose) polymerase 1 (PARP1) degradation and affect genome stability [87]. This supports binding-partner dependence, but not a universal biphasic YAP dose–response relationship. Verteporfin also has YAP-independent cytotoxic effects [88], making genetic perturbations important for interpreting pharmacological findings. Whether upstream mechanosensing ultimately affects DDR depends additionally on how the nuclear envelope transmits load and maintains transport and compartmentalization.

### 2.7. Intermediate Filaments and Microtubules in Nuclear Force Transmission

The preceding subsections follow actin, and this focus is not arbitrary: actomyosin generates most of the traction force a cell applies to its surroundings, and the perinuclear actin cap is the most direct route from adhesion to envelope. Restricting attention to actin nevertheless leaves two of the three cytoskeletal systems unaccounted for, and both are mechanically connected to the nucleus. Vimentin and keratins form networks whose material properties differ markedly from actin: they are extensible, they strain-stiffen at low strain, and they sustain large deformations without rupture, so they protect the nucleus precisely where actin transmits force most abruptly [89,90]. The connection to the envelope is direct, because nesprin-3 on the outer nuclear membrane binds plectin, which in turn binds intermediate filaments [91]. That route does not end at the outer membrane: its inner terminus is the lamina, and the two networks share more than a mechanical junction, since lamins are themselves type V intermediate-filament proteins. Vimentin or keratin loss and partial weakening of the lamina can therefore produce similar increases in nuclear deformation even though the proteins involved are distinct, which is why assays that measure deformation alone struggle to assign a phenotype to one network, and why the two are best perturbed separately when the question concerns anchorage rather than force transmission.

Functional evidence is accumulating in both directions. Keratins in colonic epithelial cells couple to the envelope and help maintain its integrity, and keratin 8 loss compromises nuclear envelope integrity [92]. Perinuclear assembly of vimentin induces nuclear dysmorphia in cancer cells, linking intermediate filament (IF) organization to the irregular nuclear shapes long used as a pathological marker [93]. In glioblastoma, intermediate filaments promote invasion by controlling nuclear deformation and the mechanosensitive expression of MMP14, which places IFs upstream of both nuclear shape and matrix remodeling in the same tumor type discussed in Section 2.1 [94]. Together these studies indicate that IFs are not passive bystanders but set the deformation that the nucleus experiences for a given actomyosin load.

Microtubules contribute through a different logic. Rather than transmitting traction, they position and pull the nucleus: nesprin-4 recruits kinesin-1 to the outer nuclear membrane, and microtubule motors generate forces that move the nucleus and, in migratory cells, orient it relative to the leading edge [91,95,96]. The same machinery is used during the DNA damage response. Damage-site capture by nuclear-envelope tubules requires DDR kinases, ATAT1-dependent microtubule acetylation, LINC components, NUP153, the lamina, and kinesin acting together [97], and 53BP1–LINC–microtubule coupling drives the movement of damaged chromatin in BRCA1-deficient cells [98]. Microtubule-dependent forces therefore operate both on the resting nucleus and on lesions after damage, and they are the most plausible route by which nuclear position—as distinct from nuclear stiffness—enters radiation biology. This route remains largely unexplored in irradiated tumor cells: no study we identified has combined microtubule perturbation, measured nuclear position or strain, and clonogenic survival after irradiation.

Section 2 has followed force from the matrix to the cell surface and then through all three cytoskeletal systems, but it stops at the nuclear envelope. Every route described above changes what the nucleus experiences, and none of them specifies what the nucleus does with that input. The envelope is not a passive recipient in this sequence: it bears the load, sets what crosses it, and can fail under it, and each of those functions has a different relationship to DNA damage processing. Section 3 takes up the envelope on those terms, treating force transmission, transport, pore dilation, barrier restoration, and remodeling as separable processes rather than as one property called nuclear stiffness.

## 3. The Nuclear Envelope as a Mechanosensitive Signaling Hub

The mechanisms described in Section 2 transmit extracellular and membrane-level mechanical information toward the nucleus through adhesion complexes, actomyosin contractility, ion-dependent signaling, and transcriptional adaptation. The nuclear envelope is the next control point in this pathway. Its lamina, LINC complexes, nuclear pore complexes, and repair systems determine how force changes nuclear shape, transport, compartmentalization, and damage-site organization. Chromatin compaction is itself the largest single mechanical component of the nucleus, but its regulation belongs to the DNA damage response and is treated in Section 4.2; what concerns us here is the envelope that contains it. One further property of the same compartment, its hydration state, is added because it shifts within minutes of an osmotic change and is easily mistaken for a lamina property in assays that do not control it. The nucleolus, which is the largest mechanical inhomogeneity inside the nucleus and one of its most radiation-sensitive structures, is treated with the radiation response in Section 4.7 rather than here. Evidence from these systems does not yet establish a universal mechanical predictor of radiation response. It instead defines the nuclear processes that could connect tumor mechanics with DNA damage processing and cell fate.

### 3.1. Nuclear Lamins, Nuclear Hydration, and Nuclear Mechanical Properties

A-type and B-type lamins form interacting but incompletely overlapping networks that contribute to nuclear shape, peripheral chromatin anchoring, and transcriptional organization [99,100,101,102]. Lamina function depends on composition, spatial organization, processing, and chromatin coupling, rather than abundance alone. Lmna loss increases nuclear strain under load [29], while adaptation of lamin A to material conditions and isoform-specific contributions to nucleocytoskeletal coupling demonstrate structural tunability [30,71]. Because the two lamin families occupy different regions of the same network, an intervention that removes one rarely leaves the other functionally intact, and this is a common source of apparent contradictions between lamin studies.

Precursor processing is a separate variable. C-terminal processing of prelamin A produces mature lamin A; ZMPSTE24 deficiency can cause prelamin A accumulation and abnormal nuclear structure [103]. In classical Hutchinson–Gilford progeria syndrome, altered LMNA splicing produces progerin, which lacks part of the C-terminal sequence and disrupts normal maturation [104]. These mechanisms are not captured by total lamin A/C abundance, and progeria findings cannot be directly extrapolated to tumor radiation response.

Nuclear stiffness, deformability, mechanical resilience, and barrier integrity describe different properties. Harada et al. showed that increasing lamin A relative to B-type lamins raises the barrier to confined migration, whereas excessive softening can compromise stress survival [105]. Chromatin and lamin A contribute at different amplitudes and rates of deformation, so a modulus obtained at one setting is not a nucleus-wide constant [106]. Consequently, identical protein abundance or a single atomic force microscopy (AFM) measurement cannot determine whole-nucleus behavior under different loads.

One determinant of nuclear mechanics is rarely reported at all: water. The nucleus is a hydrated, crowded compartment, and its stiffness and volume respond within minutes to osmotic shifts. When osmotic stress is applied to chondrocytes and other cells, nuclear volume, chromatin condensation, and the mechanical response of the nucleus change together, and the changes are large enough to be confused with genuine cytoskeletal or lamina effects [107]. More recently, nucleocytoplasmic density has been shown to be actively homeostatically regulated and conserved across eukaryotes, which implies that water and macromolecular content are controlled variables rather than passive consequences of transport [108]. This matters practically for a literature built largely on in vitro mechanical measurements: culture medium osmolarity, fixation, and the hydration state of tumor tissue at the time of measurement all shift the readout. Edematous and necrotic regions of a tumor differ substantially in water content, as they do in collagen density, and we are not aware of a radiotherapy study that has measured the two together. Hydration should therefore be recorded as a covariate whenever nuclear stiffness is proposed as a stratifying variable.

Lamins additionally contribute to DDR through protein stability and complex organization. A-type lamin deficiency can alter 53BP1 availability, ATM-related responses, and BRCA1/RAD51 repair modules [40,41,42]. These experiments connect nuclear structural proteins to damage processing, but do not necessarily isolate mechanical effects from protein binding, transcription, or degradation. Nuclear mechanics is therefore not the default explanation for every lamin-associated DDR phenotype.

Lamin B1 further illustrates the non-monotonic relationship between abundance and function. Liu et al. found that lamin B1 depletion affects RAD51 stability, recruitment, and post-irradiation survival [109]. Conversely, Etourneaud et al. showed that excess lamin B1 sequesters 53BP1 in complexes away from damage, impairing 53BP1 recruitment and increasing chromosome abnormalities [43]. These perturbations affect different repair steps and cannot be reduced to greater lamin abundance conferring greater resistance. The relevant variable is therefore not lamin abundance alone, but the combination of lamina composition, processing state, chromatin coupling, deformation regime, and damage-factor organization. This distinction leads to the next level of analysis: how force is transmitted between the cytoskeleton and the nuclear envelope.

### 3.2. LINC Complex-Mediated Force Transmission

The LINC complex connects the nucleus and the cytoskeleton through inner nuclear membrane Sad1/UNC-84 homology (SUN) proteins and outer nuclear membrane Klarsicht/ANC-1/Syne homology (KASH)/nesprin proteins. Giant nesprin-1/2 isoforms can link actin, nesprin-3 connects intermediate filaments through plectin, and nesprin-4 associates with kinesin and microtubules [91,95,96]. These different connections mean that perturbing one SUN protein or nesprin does not represent all LINC functions—the complex is a family of parallel mechanical routes rather than a single conduit, a point that also bears on the intermediate-filament and microtubule pathways discussed in Section 2.7. For the intermediate-filament route in particular, the inner terminus described in Section 2.7 is the lamina considered above, and the relationship is closer than contact: lamins are themselves intermediate-filament proteins, so nesprin-3–plectin coupling joins two networks of shared structural ancestry. Epithelial cells add a mode of nuclear anchorage, in which keratins inserted into desmosomes and hemidesmosomes hold the nucleus in position within a loaded tissue. LINC perturbation can affect both force transmission and nuclear anchorage.

Using dominant-negative KASH/SUN constructs, microneedle pulling, and substrate stretching, Lombardi et al. directly quantified cytoplasmic displacement and nuclear strain, showing that LINC disruption weakens cytoplasm-to-nucleus force transmission [26]. Some mechanically induced gene responses nevertheless persisted despite reduced nuclear deformation, indicating that direct deformation and other mechanosensing pathways can be separated experimentally—an early demonstration that force transmission and mechanotransduction are not the same thing.

SUN proteins also participate in DDR through mechanisms that are not purely mechanical. Sun1/Sun2 double-deficient fibroblasts exhibit cell-cycle, spontaneous-damage, and peripheral-chromatin abnormalities, and SUN1/2 interact with the DNA-dependent protein kinase (DNA-PK) complex [110]. Component loss also changes localization, chromatin, and complex assembly, so these results do not independently identify the contribution of trans-envelope load. Perturbations that preserve expression while changing load-bearing connections can help separate mechanical and scaffolding functions, and such separation-of-function alleles would be valuable reagents for the experiments proposed in Section 7.

LINC can organize damaged chromatin movement. Lottersberger et al. implicated 53BP1, SUN1/2, and microtubules in the mobility of deprotected telomeres and a subset of radiation-induced double-strand breaks (DSBs); corresponding nesprin-4/kinesin evidence mainly involved telomere-joining experiments [98]. In BRCA1-deficient cells under PARP inhibition, this 53BP1-dependent, LINC-mediated mobility of damaged chromatin can increase inappropriate end encounters.

The interpretive rule that follows is narrow but important: movement must be matched to the damage substrate and the intervention, because greater mobility does not imply more accurate repair and can, in some settings, increase misjoining. LINC-dependent force transmission can change nuclear deformation and damage-site mobility, but the resulting effect on repair depends on the type of lesion, the available repair pathway, and the cellular context. These distinctions also raise a transport question: mechanical changes in the nuclear envelope may alter which regulatory and repair factors enter, leave, or remain within the nucleus.

### 3.3. Mechanoregulation of Nuclear Pore Complexes: Transport, Dilation, and Shuttling

Nuclear deformation and envelope tension can affect nuclear pore complex (NPC) transport. Elosegui-Artola et al. combined substrate stiffness, cytoskeletal perturbation and direct nuclear force to show that flattening and mechanical coupling lower the transport barrier for YAP and promote its nuclear entry [27], linking force to a specific functional cargo rather than to all DNA repair proteins. Andreu et al. used reporters differing in size and nuclear localization signal to distinguish passive diffusion from receptor-mediated transport under controlled nuclear force [28]. Mechanical input alters both processes, but net accumulation also depends on cargo size, receptor binding and retention, so these results support force-sensitive selective transport without implying a shared Ran-dependent mechanism or a common direction for all cargoes.

The pore itself is mechanically plastic, which is distinct from envelope rupture. Individual NPCs dilate and contract, and the dilation state modulates permeability and fluid flux, so treating the NPC as a fixed channel underestimates how much transport can change under load [111]; pore diameter and nucleoporin arrangement adjust to the tensile state of the envelope [112]. Dilation and rupture are easily conflated, because dilation is reversible and does not breach the barrier while increasing non-selective flux that can register as leakage in a reporter assay, whereas rupture is a discrete failure event. Experiments that score leakage alone cannot distinguish the two, and a change in nuclear accumulation of one factor after loading is not by itself evidence of cargo-specific regulation. NPC plasticity has been proposed as a targetable axis at the intersection of nuclear mechanics and nucleocytoplasmic signaling [113], although no study has tested whether preventing force-induced dilation alters radiosensitivity.

Shuttling adds a time dimension that static localization ratios cannot capture. Directional transport depends on the RanGTP gradient, maintained by chromatin-bound RCC1 and cytoplasmic RanGAP, which sets the loading and release of importin and exportin cargoes [114]. A mechanical input can act at several points in that cycle, and the best-characterized example is cargo-specific: binding of importin-7 to YAP is modulated by mechanical tension, so force changes nuclear accumulation by acting on the transport machinery and not only on the cargo [115]. The nucleus is also viscoelastic, responding differently to how fast and how far it is deformed, because the two components relax on different time scales, so that the same nucleus behaves elastically over short intervals and viscously over long ones [106]. A brief load may therefore relax before import or export changes appreciably, whereas a sustained load can establish a new steady state that persists after unloading. Distinguishing these regimes requires time-resolved flux measurements such as photobleaching or photoactivation of a cargo in cells under controlled loading [116], not endpoint ratios; the minutes-to-hours over which repair factors must reach lesions overlap the reported relaxation times, so one post-irradiation ratio cannot separate sustained from transient redistribution.

For DDR, the unresolved question is whether mechanically regulated transport changes the available nuclear pool and damage-site function of specific repair factors. Ratios, NPC transit, intranuclear diffusion, damage-site recruitment and active complex formation are not equivalent. Cargo-specific measurements with controls for abundance and cell cycle, post-irradiation recruitment and clonogenic endpoints can test this interface; until then its radiation relevance remains a mechanistically grounded hypothesis, because the evidence supports force-sensitive transport of selected cargoes but not a general rule for all DDR proteins. Establishing a radiation effect requires one experiment to measure mechanical input, cargo-specific transport, damage-site recruitment and long-term survival. The interface also connects transport to barrier failure, because rupture redistributes proteins in a way that is not equivalent to regulated transport and, as the next subsection shows, not the same process as dilation.

### 3.4. Nuclear Envelope Rupture and Barrier Restoration

Confined migration and actin-mediated nuclear confinement can rupture the primary nucleus, disrupting compartmentalization, redistributing repair factors, and producing additional DNA damage [31,32,33,117]. Barrier-to-autointegration factor (BAF) recognizes exposed chromatin at rupture sites and cooperates with LEM-domain (LAP2–emerin–MAN1) proteins and endosomal sorting complex required for transport (ESCRT)-related membrane remodeling to restore the barrier [117,118,119]. Membrane continuity and DNA break repair are distinct endpoints: resealing does not automatically establish normal repair-protein localization or accurate genome repair.

Rupture frequency, duration, resealing, and recovery of protein localization jointly determine compartmental consequences. Work on LRRC59 further implicates spatial restriction of nuclear transport factors and membrane repair machinery in envelope integrity [119]. Leakage of a nuclear localization reporter identifies a barrier failure but does not fully describe recovery capacity or the subsequent DDR state.

The order of deformation, rupture, and DNA damage is not fixed. Deformation can produce replication stress, while impaired envelope integrity can permit three-prime repair exonuclease 1 (TREX1)-associated DNA processing [120,121]. In other models, increased damage within blebs is separable from detectable rupture [122]. Confined migration of developing neurons can also generate topoisomerase IIβ-related DSBs without detectable envelope rupture [123]. Reverse causation is possible: DNA damage can promote rupture through ataxia-telangiectasia and Rad3-related (ATR)-mediated lamin A/C phosphorylation [124]. Time-resolved measurements are therefore needed to identify initiating events. This temporal distinction is important for interpreting radiation studies: a pre-existing mechanical defect may alter the response to irradiation, whereas irradiation-induced damage may later weaken the envelope and promote rupture, and measurements taken only after irradiation cannot reliably distinguish the two directions of causality.

Micronuclei commonly arise from chromosome segregation errors or chromosome fragments and differ from primary nuclei in envelope assembly, pore distribution, and replication competence. Liu et al. linked abnormal envelope assembly to subsequent micronuclear damage [125]; Hatch et al. observed persistent envelope collapse, and Zhang et al. connected micronuclear damage to chromosome fragmentation and chromothripsis [126,127]. Micronuclear failure consequently has a distinct formation history and pathological consequence, rather than being a miniature equivalent of transient primary nuclear rupture.

Barrier failure also increases opportunities for DNA to encounter cytosolic sensing systems, but recruitment of cyclic GMP-AMP synthase (cGAS), its catalytic activation, and downstream stimulator of interferon genes (STING) signaling are separable [128,129,130]. Section 5.1 takes up how this connects to post-irradiation cell fate rather than treating rupture as a single upstream switch. The nuclear envelope can therefore affect radiation response through at least five connected but distinct processes: force transmission, selective transport, pore dilation, barrier restoration, and damage-induced remodeling. Rupture positivity or pore dilation alone cannot define a fixed sensitive or resistant state.

Taken together, this section identifies the nuclear envelope as a physical-genomic interface rather than a passive boundary. The five processes listed above are not parallel readouts of one underlying variable. Force transmission, selective transport, pore dilation, barrier restoration, and damage-induced remodeling each fail in a different way and each carries a different implication for DNA processing. Lamina composition and processing state, and the hydration state of the compartment, add further axes along which otherwise similar nuclei can differ, and those axes can move in opposite directions under the same load. Which axis a given assay reports therefore determines how its result should be read, and that is the question the framework in Section 6 is built to address. What this section has not established is whether any envelope property alters the processing of radiation-induced lesions rather than their formation. Section 4 takes up that question directly, beginning with the lesions themselves.

## 4. Mechanical Regulation of the DNA Damage Response

Radiation response depends not only on the lesions produced, but also on how those lesions are processed. This section examines that process in sequence: from damage type and chromatin context to signaling, repair-factor recruitment, damage-site movement, and pathway use. Cell-cycle position runs through all of these steps and is treated separately in Section 4.6, because it is simultaneously a determinant of mechanical state and a determinant of repair capacity. Throughout, damage markers, repair activity, repair fidelity, and long-term survival are treated as distinct outcomes.

### 4.1. Radiation-Induced DNA Damage in a Mechanical Context

Ionizing radiation produces base lesions, single-strand breaks, and double-strand breaks, with some lesions clustered in space. Track structure, dose, and radiation quality influence complexity; high linear energy transfer (LET) radiation generally increases the proportion of locally clustered and difficult-to-repair damage [131,132]. Damage and survival can also vary with depth along a proton beam [133]. LET alone does not capture every track characteristic, and low-LET photon findings cannot directly represent distal proton or carbon-ion conditions—an important limit when mechanical phenotypes are proposed as general predictors across modalities.

Nuclear structure could influence subsequent lesion processing through chromatin accessibility, factor supply, compartmentalization, and spatial rearrangement without requiring a general matrix effect on initial energy deposition. Complex lesions demand additional end processing and local remodeling, making them useful for testing whether the nuclear environment limits repair [131,132]. Comparing initial and residual damage across mechanical conditions helps distinguish differences in damage formation from differences in damage clearance, and this comparison is more informative than either measurement alone.

Ser139-phosphorylated histone H2AX (γH2AX) and 53BP1 foci are damage-associated chromatin signals rather than one-to-one DSB counts. Focus clustering, dose nonlinearity, and ATM/DNA-PK signaling contributions affect their kinetics [134,135,136]. A focus-based damage yield measured in cells whose chromatin state has been manipulated is therefore confounded, and in a direction that matters for the argument that follows. Engineered repair reporters measure completed events on particular substrates, whereas colony formation measures long-term reproductive capacity [137,138]. Joining products and chromosome abnormalities provide additional information about fidelity. These distinctions define how the following evidence should be read: a mechanical effect on a damage marker is not equivalent to an effect on recruitment, on pathway use, on joining fidelity, or on long-term survival. The next step is to consider the chromatin environment that determines how lesions and repair factors are organized.

### 4.2. Chromatin Organization, Compaction, and Damage Accessibility

Lamina-associated domains and peripheral heterochromatin anchoring connect the nuclear envelope to genome organization [100,101,102]. The consequence for repair is directional: breaks in these domains are repaired predominantly by ligation-based joining, are constrained to the periphery and fail to migrate inward to recombine with homologous sequences, so peripheral anchoring biases the pathway available to a lesion alongside differences in damage signaling [139]. Chromatin also contributes to nuclear mechanics, with distinct contributions from chromatin and lamin A at small and larger deformations [106]; whole-nucleus modulus, local chromatin accessibility and damage-site remodeling are therefore not interchangeable. Goodarzi et al. showed that processing a subset of heterochromatin-associated DSBs requires ATM signaling and KAP1-related remodeling, and subsequent work implicated regulation of the CHD3 nucleosome remodeler [140,141]. These experiments establish context-dependent repair requirements rather than direct effects of external material manipulation, and cell stretching has since connected mechanical stress to ATM-related cytoskeletal and chromatin remodeling [142].

Compaction has a physical consequence that operates before any repair consideration arises: when chromatin is condensed, the probability that a radiation track produces a DNA breakfalls [143]. The magnitude varies with chromatin context and lesion class, so compaction is better treated as one modifier of lesion yield than as a fixed protection factor. Because this is a lesion-formation effect rather than a repair effect, chromatin state must be controlled before any claim about mechanical regulation of repair can be sustained; it carries no implication about repair capacity, which is the subject of Section 4.3, Section 4.4, Section 4.5 and Section 4.6. The same logic applies in reverse during loading: heterochromatin behaves as a mechanoprotective buffer, and failure of adaptive nuclear softening converts a normally tolerated strain into DNA damage without any change in exogenous radiation [44].

Cyclic mechanical stress reduces ultraviolet (UV)-induced DNA damage in a loading-dependent manner [144], whereas brief cyclic stretch alters chromatin accessibility and supports protection against mechanically induced DNA damage [145]. UV-induced lesions include pyrimidine dimers repaired by nucleotide excision repair, whereas ionizing radiation produces a different lesion spectrum; neither study establishes protection against therapeutic ionizing radiation. These findings complicate the intuition that mechanical stress is uniformly damaging: chromatin appears to have a mechanically accessible state in which damage formation is reduced and repair favored, and that state can be reached by prior loading. Whether an equivalent state can be induced before radiotherapy, and whether it would protect tumor and normal tissue equally, is unknown, and the preclinical logic would require a therapeutic window rather than merely a reduction in damage.

A three-dimensional pancreatic ductal adenocarcinoma (PDAC) bioprinting study connected stiffer gelatin methacryloyl materials, glycolysis/lactate, histone H3 lysine 18 lactylation (H3K18la), and RAD51 expression [146]. Interventions using 2-deoxyglucose or lactate, chromatin immunoprecipitation, RAD51 knockdown, and colony formation after 4 Gy irradiation provide mechanistic support beyond association alone. Material formulation, metabolic intervention specificity, and the independent contribution of H3K18 modification nevertheless remain to be separated. These measurements do not directly quantify nuclear modulus or repair fidelity, and the lactylation intermediate is a metabolic readout rather than a mechanical one.

Together, these findings connect mechanical conditions with chromatin and metabolic regulation, but they do not support a general rule based on bulk chromatin condensation. Effects depend on chromatin region, lesion type, cell cycle, and local remodeling. Chromatin relaxation cannot universally be equated with improved repair, nor can compaction be taken to imply HR suppression or NHEJ enhancement; pathway use requires appropriate substrates and functional assays.

Chromatin state is only one part of the repair environment. The next question is whether mechanical conditions alter damage signaling, the availability of repair factors, or their function at damage sites—three possibilities that are frequently conflated and require different assays to separate.

### 4.3. Damage Signaling and Repair Factor Recruitment

ATM, ATR, and the catalytic subunit of DNA-dependent protein kinase (DNA-PKcs) are kinases, whereas PARP1 participates in DDR through ADP-ribosylation; these enzymes differ in substrates and activation contexts [7,147,148]. γH2AX is a phosphorylated histone mark directly recognized by MDC1, and RNF8-mediated ubiquitin signaling organizes downstream DDR proteins [149,150]. Damage-site arrival, maintenance of an available nuclear protein pool, and active complex formation therefore require separate analysis.

In tunable stiffness systems, Deng et al. found that low stiffness promotes ubiquitin Thr66 phosphorylation through RAP2–MAP4K4/6/7, affecting RNF8-mediated ubiquitin chain formation and downstream 53BP1/BRCA1 assembly [13]. Early γH2AX, MDC1, and RNF8 recruitment did not decline to the same extent as downstream factors, separating localization from enzymatic signaling function. DR-GFP and EJ5-GFP reporter activity, residual damage, and survival measurements support functional relevance. However, the extent of validation differs among cell lines, and HEK293 results do not directly represent patient tumors.

In HCC, Wang et al. connected greater stiffness to NEDD4-related PFKFB3 stabilization, post-irradiation PFKFB3 nuclear translocation, and Ku70 interaction [12]. Growth/viability, γH2AX, and neutral comet measurements across 6, 10, and 16 kPa conditions support a stiffness-associated damage-processing mechanism that is comparatively well developed. The animal experiments compared growth of pre-irradiated or unirradiated cells implanted into different hepatic backgrounds, rather than local radiotherapy of established tumors; these endpoints also do not independently establish enhancement of accurate c-NHEJ.

Li et al. showed that SDC4 and PKD2 cooperate in mechanosensing in oxaliplatin-treated hepatocellular carcinoma cells, promoting protein phosphatase 2A (PP2A) assembly and activity, increasing BANF1 nuclear translocation, and supporting MRE11-RAD50-NBN (MRN) complex formation and downstream signaling [151]. This connects mechanosensing molecules to DDR assembly, and the sequential perturbation design helps localize the functional step beyond observing damage foci alone. Mechanical mediation measurements are still needed to distinguish effects of nuclear load from parallel biochemical pathways.

Direct mechanical ATM responses differ from canonical DSB responses. Bastianello et al. found that cellular deformation activates ATM through cytoskeletal and reactive oxygen species-related processes independently of its canonical MRE11 activation step, alongside cytoskeletal and chromatin changes [142]. This implicates ATM in mechanical stress, but does not by itself establish improved repair fidelity or clonogenic survival after therapeutic irradiation.

Canonical ATR sensing involves replication protein A (RPA)-coated single-stranded DNA and replication stress [147]. Under osmotic stress, Kumar et al. observed an ATR-associated nuclear envelope checkpoint, including ATR-dependent CHK1 phosphorylation [152]; therefore the response was not devoid of canonical downstream signaling. In a different setting, ATR-mediated lamin A/C phosphorylation after DNA damage can promote envelope rupture [124]. These experiments reveal stress responses and damage-induced structural remodeling, respectively, rather than a single demonstrated pathway from external load to improved radiation repair.

DNA-PKcs contributes to c-NHEJ, with nuclear envelope connections involving SUN1/2-associated complexes and early damage signaling [110,136]. Shokrollahi et al. found that DDR kinases, ATAT1-dependent microtubule acetylation, LINC, NUP153, the lamina, and kinesin cooperate in forming DSB-capturing nuclear envelope tubules (dsbNETs) [97]. These structures support break processing and show that damage signaling can actively organize a nucleocytoplasmic mechanical response; what they do not show is whether external mechanical conditions before irradiation determine that response.

PARP1 can support XRCC1 assembly or stabilization at oxidative DNA damage sites [148]. In cardiomyocyte stress experiments, Rajamohan et al. observed increased PARP1 acetylation and activity with SIRT1-related regulation, including under cyclic mechanical stretch [153]. This is biochemical evidence associated with mechanical stress in nonmalignant cells, not independent proof of radiation repair or nuclear mechanical mediation. Rather than rank the mechanosensitivity of ATM, ATR, DNA-PKcs, and PARP1 across different models, we ask how each enzyme is activated, localized, assembled into functional complexes, and linked to repair outcomes.

Signaling and factor recruitment determine which repair activities are available at a lesion. They do not, on their own, explain how damaged regions encounter one another. Section 4.4 therefore considers nuclear actin and DSB mobility, while distinguishing movement from accurate joining.

### 4.4. Nuclear Actin and Double-Strand Break Mobility

Signaling at the lesion is one determinant of repair; where the lesion sits and how it moves is another. DNA damage itself can induce nuclear actin assembly. Belin et al. linked Formin-2/Spire-associated F-actin formation to residual damage [154], while subsequent work showed that DYRK1A-mediated Spir1 phosphorylation regulates F-actin on damaged chromatin and repair [155]. These structures are initially DDR effectors; the involvement of cytoskeletal components alone does not establish an external mechanical input.

Different breaks have different spatial requirements. Schrank et al. showed that ARP2/3 supports clustering of HR-permissive breaks in mammalian S/G2 cells [156], while Caridi et al. connected nuclear F-actin and myosin to controlled relocation of heterochromatic breaks in Drosophila and mouse cells [157]. These findings concern particular chromatin regions and repair contexts, rather than a common movement requirement for every radiation-induced DSB.

Movement can also increase inappropriate joining. In BRCA1-deficient cells under PARP inhibition, 53BP1–LINC–microtubule-associated motion can promote encounters between incorrect ends [98]. Faustini et al. further used Lmna- and lipid metabolism-related perturbations to connect envelope invagination/deformability, microtubule-dependent break movement, and PARP inhibitor cytotoxicity [158]. This drug and repair-deficiency context cannot substitute for randomly distributed radiation damage, and displacement, joining accuracy, and clonogenic survival require separate evaluation. The gap in this literature is one of input rather than of mechanism. No study we identified has manipulated an external mechanical variable—substrate stiffness, applied strain, or confinement—and then measured break mobility, joining accuracy, and survival in the same cells. The mobility literature is DDR-intrinsic: it establishes what the nucleus can do with its own cytoskeleton, and its relevance to tumor mechanics remains an inference by analogy with Section 2.7 rather than a demonstrated link.

Thus, nuclear actin and LINC-dependent movement can affect the location and encounter of damaged regions, but the outcome depends on lesion type, repair capacity, and cellular context. Whether movement changes pathway use must be distinguished from whether it increases accurate repair.

### 4.5. Mechanical Regulation of Repair Pathway Choice

Utilization of HR, c-NHEJ, and alternative end joining depends on cell cycle, end resection, template availability, and break structure [6,159,160]. Polymerase θ-mediated end joining (TMEJ) is a major alternative joining mechanism that can use microhomology and compete with recombination, but it does not encompass every alternative joining event [161,162]. HR is not the default route for all G2 breaks, and c-NHEJ is not uniformly erroneous.

A change in pathway choice requires evidence of altered relative pathway use, not merely parallel changes in multiple repair readouts. Low stiffness in the study by Deng et al. reduced both HR and overall end-joining reporter activity, consistent with a shared upstream signaling limitation [13]. EJ5-GFP is not specific for c-NHEJ and cannot independently establish a switch from HR to classical joining [137]. Likewise, PFKFB3–Ku70 interaction supports involvement of a repair module, but γH2AX or comet kinetics do not establish increased accurate c-NHEJ [12].

It is worth stating how a mechanical variable could bias the HR/NHEJ decision, because the question has a narrower answer than is sometimes assumed. Chromatin mobility is one plausible route. HR-permissive breaks are actively clustered by nuclear actin and myosin, whereas c-NHEJ can proceed on relatively immobile ends, so any constraint on movement, such as a rigid lamina, an over-crosslinked chromatin network, or a stiff substrate that limits nuclear deformation, could alter repair-pathway use, depending on lesion type and cellular context [98,156,157]. The same route has a static counterpart: breaks anchored at the nuclear periphery are repaired by ligation, whereas breaks in the nuclear interior engage recombination, so nuclear position itself biases pathway choice, and conditions that reshape lamina-associated domains could alter that bias [139]. The 53BP1/BRCA1 balance operates on a different logic, independent of movement. 53BP1 accumulation at breaks favors c-NHEJ, and mechanical state feeds into this balance through RNF8-dependent ubiquitin chain formation [13], A-type lamin-dependent 53BP1 availability [40,41,42], and lamin B1 sequestration of 53BP1 away from lesions [43]. Factor supply acts at a third point, through altered transport or pore dilation (Section 3.3), and can limit resection machinery without affecting damage recognition at all.

No study has yet measured pathway use as a relative quantity, combining reporter ratio with joining-product sequence profiles, under a controlled mechanical perturbation, at a defined cell-cycle stage, with an independent fidelity endpoint. Until such a study exists, the most defensible statement is that mechanical state plausibly shifts the competition between resection-dependent and ligation-based repair, and that the current evidence consists of component observations rather than a demonstration of pathway switching. The experiments needed are specified in Section 7.1.2.

Mechanical state could affect pathway utilization and fidelity through chromatin accessibility, the 53BP1/BRCA1 balance, factor supply, and damage mobility. Separating these possibilities requires matched cell cycle and initial damage, together with pathway reporters, insertion/deletion and microhomology profiles of joining products, and chromosome translocation measurements; such combinations distinguish accurate repair from faster misjoining or altered damage signaling alone.

### 4.6. Cell-Cycle State as an Integrating Variable

Cell-cycle position is the most consistent single determinant of radiosensitivity [163], and it is also a determinant of nuclear mechanical state; the two facts are usually treated separately, but they are not independent. Repair pathway availability is governed by cell-cycle phase. HR requires a sister chromatid and is therefore largely confined to S and G2, whereas c-NHEJ operates throughout the cycle [6], so mechanical effects on pathway use may be partly mediated by changes in cell-cycle distribution. That distribution is itself mechanically sensitive. Substrate stiffness and spreading alter cycle progression through YAP/TAZ and cyclin-dependent kinase inhibitors, which means that a stiff substrate can change radiosensitivity simply by shifting the fraction of cells in G2/M, without any direct effect on repair chemistry.

Pathway utilization is often summarized as an S/G2-versus-G1 dichotomy, but that summary is a simplification. Within G2, repair competence declines as cells approach mitosis, and mitotic chromosome condensation is accompanied by attenuated damage signaling and reduced mobility of damage sites, so a ratio measured in an asynchronous population averages over substates with different repair capacity. We therefore require that the substage at which pathway use is measured be specified wherever the marker permits (Section 7.1.1 and Section 7.1.2), and we do not attribute a shift in pathway ratio to mechanical conditions unless the cycle distribution was measured in the same experiment.

More directly, the nucleus is mechanically different at each cycle stage. Nuclear and actin mechanics change measurably from G1 to G2, and these changes affect nuclear integrity under load; loss of peripheral H3K9me3 contributes to nuclear softening from G1 to G2 [164]. Separately, Swi6 condensation contributes to nuclear stiffness in fission yeast [165]. A mitotic cell rounds up, and its envelope is dismantled and reassembled, and its nucleus experiences forces that have no counterpart in interphase—yet mitotic cells are also the most radiosensitive. That sensitivity is associated with attenuated DNA damage signaling and repair during mitosis, so lesions are expressed as chromosome aberrations rather than repaired; it is not evidence of a higher lesion yield, and the compaction argument in Section 4.2 concerns lesion formation rather than the fate of a lesion once formed. A mechanical measurement made on an asynchronous population therefore averages across states that differ in both stiffness and radiosensitivity, and an asynchronous clonogenic endpoint cannot attribute a survival difference to nuclear mechanics rather than to cycle redistribution. We regard cell-cycle-resolved nuclear phenotyping, ideally by imaging plus DNA-content labeling in the same experiment, as a minimum requirement for the claims this field wishes to make, and we return to this point in Section 7.2.

### 4.7. Nucleolar Stress and Ribosomal Surveillance

The nucleolus belongs in a discussion of damage processing for a reason that is easy to overlook: many phenotypes attributed to the lamina after irradiation, including nuclear size, chromatin texture, and apparent envelope irregularity, are partly nucleolar. It is also the largest mechanical inhomogeneity inside the nucleus and one of its most radiation-sensitive structures. It is not a membrane-bound organelle but a phase-separated condensate whose material state, viscoelastic with RNA flowing through it advectively, determines both its form and its output in ribosome biogenesis [166]. Its assembly is maintained by weak multivalent interactions rather than by a scaffold, so it responds to crowding, ionic conditions, and ATP in ways that leave the lamina unaffected.

Radiation reaches this compartment through several routes. The best-defined is transcriptional repression: chromosome breaks activate ATM, which inhibits RNA polymerase I, so ribosomal RNA synthesis falls within minutes of irradiation [167]. Breaks within the ribosomal DNA repeats recruit a specialized response that includes ATR signaling alongside the canonical machinery [168], and nucleolar morphology and composition reorganize as a consequence [169,170]. Nucleolar disruption can stabilize p53 and induce downstream transcriptional responses [171]. The nucleolus therefore sits upstream of the p53 arm of the damage response rather than downstream of it.

The mechanical route into the same compartment is less established, but it is not speculative. Changes in nuclear shape and chromatin organization alter nucleolar position and its association with heterochromatin, and that association is what keeps a large fraction of ribosomal DNA silent; loss of the nucleolar histone chaperone NPM1 is sufficient to rearrange heterochromatin [172,173]. Because the silencing state determines how much ribosomal DNA is available to be damaged and how accessible it is to repair, a mechanical input that repositions the nucleolus could in principle change both lesion formation and repair within the repeats. What distinguishes the nucleolus from every other structure discussed in Section 3 is that its assembly is thermodynamically rather than structurally maintained, so load can reach it by routes that leave the lamina unchanged.

Two implications follow. The first is a caution: radiation-induced nucleolar reorganization can mimic lamina phenotypes in assays that do not use nucleolus-specific markers, so nuclear size or chromatin texture alone should not be assigned to the envelope. The second is an opportunity: nucleolar state integrates transcriptional, mechanical and damage signals and is measurable with existing reagents such as NPM1 or fibrillarin labeling together with markers of ribosomal DNA transcription, so it could contribute to the composite phenotypes proposed in Section 7.3.1. Whether mechanical loading changes nucleolar radiation sensitivity in tumor cells has not been tested, which makes it one of the more tractable open questions in this field.

Overall, the evidence supports several possible links between mechanical state and damage processing, but it does not establish a universal sequence from matrix stiffness to a particular repair pathway or survival outcome. What the section does establish is narrower and more useful: each step at which mechanics could act has been measured in at least one system, and at each step the measured quantity, whether lesion number, focus kinetics, factor recruitment, reporter activity, or joining fidelity, answers a different question. The nucleolar response described in Section 4.7 adds a compartment in which transcriptional and damage signals converge before any repair decision is taken. None of this yet connects a mechanical state to the fate of an irradiated cell, because fate is decided by processes that run after damage processing and, in vivo, by cells that are not the tumor cell itself. Section 5 turns to those two layers: the immune consequences of envelope failure, and the death programs that determine what happens to a cell whose damage is not repaired.

## 5. Nuclear Envelope Damage, Innate Immunity, and Cell Fate

Section 4 focuses on how mechanical state may affect DNA damage processing. A separate consequence arises when nuclear or micronuclear barriers fail: DNA may become accessible to sensing systems. A third consequence operates at the tissue level, when dying cells release their contents and the immune system interprets the result. Whether either access or release leads to immune signaling, and whether that signaling changes radiation outcome, depends on DNA processing, tumor–host communication, and the time scale and endpoint being measured.

### 5.1. Nuclear and Micronuclear Rupture and cGAS–STING Signaling

Envelope failure increases opportunities for genomic DNA to encounter DNA-sensing systems. Mackenzie et al. linked envelope-defective micronuclei to cGAS sensing, while Harding et al. connected post-damage mitosis and micronucleus formation to delayed inflammatory output in irradiated cells [128,129]. These findings support abnormal chromatin compartments as signal sources, without implying that transient primary nuclear rupture invariably activates STING immediately.

Several separable steps intervene between cGAS localization and a completed response: DNA contact, catalytic production of 2′3′-cyclic GMP–AMP (2′3′-cGAMP), activation of STING, TANK-binding kinase 1 (TBK1), and interferon regulatory factor 3 (IRF3), interferon-stimulated gene expression, and host immune effects [128,129,130,174,175]. Takaki et al. observed micronuclear cGAS localization without corresponding downstream output in the irradiation, replication stress, or chromosome missegregation models examined [130]. Sato and Hayashi found that γ-ray-associated STING activation could be uncoupled from micronucleus formation, with evidence implicating mitochondrial DNA [176]. Micronucleus abundance and cGAS positivity therefore do not, by themselves, predict the strength of downstream signaling.

Chromatin state helps explain these differences. Pre-existing chromatin organization can influence cGAS recruitment to ruptured micronuclei [177]; nucleosome binding can suppress cGAS activation, and TREX1 can reduce accessible DNA [178,179]. Binding of MRN to nucleosome fragments can also release sequestered cGAS and promote DNA sensing [180]. Experiments combining BAF perturbation with mitotic checkpoint inhibition additionally linked enhanced signaling to unstable chromatin bridges [181]. Together, these findings indicate that DNA origin, processing, and packaging influence whether exposure leads to signaling—the same variables that determine whether a rupture is repaired without consequence.

Tumor-cell and host sensing are distinct. Immunocompetent mouse studies implicate host STING and type I interferon in some radiation-associated antitumor responses [174]. Carozza et al. established paracrine immune effects through perturbations of cancer-cell cGAMP export, extracellular degradation, and host sensing [175]. Tumor cGAS, tumor STING, host STING, and extracellular signal transfer represent different steps. Imported cGAMP can act on recipient-cell STING, whereas interferon signals through its receptor. A single increase in tumor-cell IFNB1 does not independently demonstrate host adaptive immune involvement.

Envelope failure thus creates a possible route to DNA sensing, but it does not determine the downstream immune outcome. The next question is how dose, DNA clearance, and observation period shape the consequences for radiation response—and how the same dying cells signal through routes that do not require cGAS at all.

### 5.2. Context-Dependent Consequences for Radiation Response

Beyond sensing, particular lamina or repair defects can increase residual damage and reduce long-term reproductive capacity after irradiation [40,109]. Surviving populations may subsequently undergo selection and adaptation; in models of persistent chromosomal instability, cytosolic DNA/STING-associated inflammation can promote metastasis [182]. The latter cannot retrospectively establish intrinsic radioresistance of the starting cells, because acute damage-processing failure and long-term tumor evolution have different causal starting points and observation windows.

Dose and fractionation can alter immune output through DNA clearance. Vanpouille-Box et al. found that higher single doses induce TREX1 and reduce cytosolic DNA and associated immunogenicity in particular tumor models [183]. The threshold is model-dependent rather than a universal clinical boundary. Li et al. further showed that SPOP promotes TREX1 degradation, whereas USP7 stabilizes TREX1; this protein-homeostasis axis regulates cytosolic DNA clearance and cGAS–STING signaling after DNA damage [184]. Immune consequences therefore depend not only on the amount of damage generated, but also on how much DNA remains available for sensing.

cGAS–STING is one arm of a broader response. Irradiated tumor cells that die in an immunogenic manner expose calreticulin on the cell surface, release HMGB1 and extracellular ATP, and thereby license dendritic-cell activation and antigen cross-presentation, a process that requires an intact host and is therefore invisible in culture [9]. The distinction matters for the present argument in two ways. Mechanically, nuclear-envelope rupture is a plausible route for HMGB1–chromatin complexes to enter the extracellular space, which would connect a mechanical event to an immune signal rather than only to a repair outcome. Clinically, ICD is dose- and fractionation-sensitive, and the biomarkers used to detect it in patients, including circulating HMGB1, surface calreticulin on tumor cells and interferon signatures, have been proposed as pharmacodynamic readouts for radiotherapy combinations [10]. None of these markers has been linked to nuclear mechanical state, and we regard that as an unexplored but testable connection.

Two cautions apply. ICD defined by consensus criteria is a stricter category than “cell death accompanied by inflammation,” and many radiotherapy studies report the latter while claiming the former [9]. Because ICD requires host sensing, its contribution to local tumor control cannot be inferred from clonogenic survival in vitro, which is why the framework in Section 6 keeps immune output as a separate axis rather than folding it into radiosensitivity.

Acute antitumor effects involving host STING and prometastatic inflammation in chronic instability models are not contradictory: compartments, duration, and endpoints differ [174,182]. A short-term interferon increase is insufficient to infer improved long-term local control. Viability, clonogenic survival, tumor growth delay, local recurrence, and metastasis are likewise not interchangeable outcomes. Longitudinal measurements and lineage tracking can separate initial sensitivity, residual-cell selection, and subsequent adaptation.

These distinctions show why immune output cannot serve as a substitute for measuring cell fate. The final subsection separates reproductive survival from the individual death processes and post-irradiation morphologies that are often reported in its place.

### 5.3. Radiation-Induced Cell Fate and Its Nuclear Regulators

The immune layer is one determinant of outcome; the fate of the irradiated cell itself is another. Radiobiology commonly evaluates that fate through loss of long-term reproductive capacity. Colony formation directly tests whether an irradiated cell can sustain a population, but a non-clonogenic cell is not necessarily dead [138]. Apoptosis and other death mechanisms describe execution processes that do not fully correspond to reproductive survival [185]. Cross-genotype comparisons by Williams et al. similarly showed different patterns for colony formation, apoptosis, and cell cycle redistribution [186].

Genetic state can redistribute post-irradiation fates. In glioma models, Lee et al. showed that PTEN/AKT-related interventions change the balance between premature senescence and apoptosis [187]. Reduced apoptosis therefore does not directly imply increased radiation survival; interpretation requires an observation window and a long-term reproductive endpoint. Mechanical perturbations can similarly redistribute fates through accompanying cell-cycle changes without involving DNA repair mediated by nuclear mechanics.

Nuclear regulators of apoptosis occupy a position that makes them easy to misread, because several act on repair as well as on death. Survivin accumulates in the nucleus after irradiation, and this accumulation is linked to DNA damage repair rather than to its canonical mitotic or anti-apoptotic functions; depleting it delays repair and increases radiosensitivity [188]. The BCL-2 family provides a second example: deregulated BCL-2 proteins alter double-strand break repair kinetics and are candidate targets for overcoming radioresistance [189], and part of the effect is positional rather than quantitative, because BCL-2 retains BRCA1 at the endoplasmic reticulum and mitochondria and reduces its availability for homologous recombination [190]. MCL-1 localizes to sites of DNA damage and regulates the response directly [191]. The traffic runs in both directions: Ku70, a core non-homologous end joining factor, binds and sequesters Bax, and acetylation of its C-terminus releases Bax and commits the cell to apoptosis [192]. Death executed from the nucleus can also be caspase-independent, because apoptosis-inducing factor translocates from mitochondria to the nucleus after damage and produces chromatin condensation and large-scale DNA fragmentation there [193]; such death is invisible to the caspase assays often used as the apoptosis readout.

A further regulator acts through location and modification rather than abundance. p53 is held at low levels by MDM2-dependent degradation and shuttles between nucleus and cytoplasm, and its phosphorylation and acetylation state determines which promoters it occupies and whether the outcome is arrest, senescence or apoptosis [194]. The nucleolar axis described in Section 4.7 feeds this system directly, because nucleolar disruption can stabilize p53 [171]. Because TP53 status also sets G1/S checkpoint competence and determines which death program is available, it confounds both the cycle-distribution argument of Section 4.6 and the fate readouts summarized here, and it should be reported in cross-model comparisons rather than assumed constant. Whether nuclear mechanical state biases p53 toward a particular modification or compartment has not been tested, which is why we treat TP53 as an effect modifier rather than as a component of the mechanical model.

An apoptosis readout therefore cannot be used as a proxy for nuclear mechanical state. In a cell with high nuclear survivin, a mechanical perturbation that improves repair would reduce apoptosis without necessarily improving clonogenic survival, and in a cell with low BCL-2 the same perturbation might shift death modality while leaving reproductive capacity unchanged. Apoptosis measurements remain useful, but only when paired with clonogenic survival and with explicit identification of the rate-limiting regulator in the model studied.

Mitotic catastrophe involves abnormal entry into or completion of mitosis by damaged cells, rather than a single molecular death-execution mechanism [185]. Time-resolved studies in irradiated HeLa S3 cells documented sequential cell-cycle delay, abnormal chromosome condensation, and nuclear fragmentation [195]. Multinucleation, micronuclei, or giant-cell morphology alone cannot determine ultimate survival; these cells may progress to death or to prolonged proliferation arrest, which favors lineage imaging combined with clonogenic endpoints.

Senescence requires sustained cell-cycle exit and multiple state markers; one positive senescence-associated β-galactosidase measurement or short-term non-proliferation is insufficient [196]. cGAS studies support its participation in senescence and inflammatory output under some stress and irradiation conditions [197], but do not establish envelope rupture in every irradiated senescent tumor cell. Secretory programs, persistence, and possible cell-cycle re-entry also influence tissue consequences.

Autophagy is an intracellular recycling process that can support adaptation or affect death under particular conditions. Autophagosome or lipidated microtubule-associated protein 1 light chain 3 (LC3-II) accumulation alone does not establish flux or autophagy-dependent death [198]. Paglin et al. observed increased acidic compartments in irradiated cancer cells, and interfering with acidification enhanced clonogenic killing, supporting a protective role in that system [199]. In breast cancer models, Huang et al. linked autophagy perturbation to post-irradiation senescence, apoptosis, and bystander effects [200]. These fate processes can interact; nuclear state is more likely to alter their entry conditions than to specify one fixed fate.

Taken together, these observations do not support a predictable relationship between greater stiffness, repair and survival. Studies differ in intervention, model, radiation conditions and endpoint, and the nuclear properties themselves can dissociate within one cell: a nucleus may deform readily while losing barrier integrity, or retain its barrier while showing limited remodeling.

Damage foci, repair-factor localization, reporter activity, immune signaling, short-term viability, and clonogenic survival measure different processes, and none of them alone establishes a complete link between mechanical input, repair fidelity, and long-term cell fate. Two layers have now been added to the picture assembled in Section 3 and Section 4: the immune consequences of barrier failure, which depend on DNA clearance and on the host, and the death programs that determine what happens to a cell whose damage is not repaired. Neither layer is predictable from the other, and neither is predictable from a mechanical measurement. The result is a literature in which individual links are well supported and their combination is not, which is the problem Section 6 addresses.

## 6. Reconciling Conflicting Evidence: A Provisional Three-State Model of Nuclear Mechanical Homeostasis

### 6.1. Conceptual Basis of the Three-State Model

Radiation resistance has been reported in both soft and stiff environments. Soft conditions were associated with resistance in some GBM and other tumor models [20,24,51], whereas greater stiffness was associated with weaker irradiation responses or altered damage processing in HCC and PDAC models [12,23,146]. Deng et al. found that low stiffness impairs ubiquitin signaling and reduces HR and end-joining reporter activity [13]. These findings do not support a consistent effect of matrix stiffness on radiation survival. Stiffness interventions can also change ligand presentation, matrix architecture, metabolism, oxygenation, and transport [18,23,48,49,50]. The endpoints differ as well: damage foci do not directly measure repair fidelity, and reporter activity is not equivalent to clonogenic survival [134,135,136,137,138]. Cell type, genotype, cell cycle, radiation quality, and observation time further limit direct comparison.

Nuclear findings expose the limits of a soft–hard model. Loss of lamin-dependent support can increase deformation or compromise envelope integrity [29,31,117], while excess lamin B1 can limit 53BP1 mobilization [43]. Progerin models show that whole-nucleus or scaffold stiffness can increase while chromatin becomes more compliant [201]. Force transmission, barrier maintenance, selective transport, and damage-site mobility are related but distinct properties [26,27,28,98]. Neither matrix modulus nor a single nuclear-stiffness value captures them all.

Most studies have not measured matrix mechanics, nuclear behavior, repair quality, and long-term survival in the same cells under matched conditions. The key question is therefore whether different combinations of nuclear support, compartmental integrity, and remodeling capacity can explain why similar mechanical inputs produce different radiation outcomes. The three-state model below addresses this question as a testable hypothesis, not as an established explanation.

### 6.2. Three-State Model of Nuclear Mechanical Homeostasis

The model describes three proposed states: insufficient structural support, maladaptive structural constraint, and coordinated integrity and plasticity. It is currently a descriptive framework that organizes findings from different systems and generates prospective predictions, not a validated classifier or a general law linking nuclear mechanics to radiosensitivity (Figure 2). The figure re-organizes the pathway summarized in Figure 1 into three functional properties and the states produced by their combinations, rather than adding a second pathway.

The states are nonexclusive and may change with loading, recovery, cell-cycle phase, or treatment. A cell may retain an intact envelope but show restricted repair-factor redistribution, or deform readily while experiencing barrier failure. No universal thresholds or survival ranking are assumed, and applicability across tumor types, genotypes, and cell-cycle states requires testing in matched models.

### 6.3. State 1: Insufficient Structural Support

Insufficient support refers to a limited ability to maintain barrier integrity under a defined load, with high nuclear strain as the expected correlate rather than a defining feature. Lmna loss can increase nuclear strain, and confined migration can cause envelope rupture [29,31,117]. Some lamin perturbations also affect repair-factor availability and post-irradiation survival [40,109]. These findings support individual components of the proposed state, but not a complete causal sequence from weak support to defective repair and reproductive inactivation.

Prospective classification should follow the criteria in Table 1 and Section 6.6 and should be made independently of survival. Deformation is recorded as a correlate and does not enter assignment. Low lamin expression, irregular nuclear shape, or a high strain value is insufficient for assignment, and persistent damage, reproductive inactivation and survival with genomic abnormalities are outcomes rather than defining features.

### 6.4. State 2: Maladaptive Structural Constraint

Maladaptive constraint refers to a mismatch between nuclear structure and the reorganization required in a specific context; its operational form is an intact barrier with incomplete recovery after unloading (Table 1). A higher relative lamin A level can increase resistance to confined migration, and excess lamin B1 can impair 53BP1 mobilization [43,105]. Progerin models show that stiffness at the nuclear or scaffold scale need not track chromatin mechanics in the same direction [201]. These findings support measuring distinct nuclear properties, but they do not show that greater nuclear stiffness generally impairs radiation repair.

A testable prediction is that some cells will show no leakage and complete resealing yet fail to restore nuclear volume or to redistribute a specified repair factor within the defined interval. Low strain, restricted deformation and altered damage-site movement are correlates and predictions rather than criteria. Chromatin compaction, limited transport and restricted break mobility should not be assumed to occur together. Effects on clonogenic survival also remain open: altered processing may reduce reproductive capacity, whereas selection may produce a different outcome among surviving cells.

### 6.5. State 3: Coordinated Integrity and Plasticity

Coordinated integrity and plasticity refers to the proposed ability to maintain an intact barrier and to restore nuclear volume and repair-factor distribution completely within the predefined interval, while permitting the reversible deformation and reorganization a given context requires. Existing studies support individual components of barrier maintenance, chromatin remodeling and repair-factor dynamics [26,27,28,117,118,119,140,141]. They have not shown that these components form a universal optimum for repair fidelity or radiation survival.

Testing this state requires predefined measures of barrier maintenance and recovery, with deformation, chromatin remodeling and transport recorded as correlates. Faster focus resolution does not establish more accurate repair, and survival after irradiation alone does not demonstrate coordinated integrity and plasticity.

### 6.6. Operational Definitions and Discriminating Experiments

A model that cannot be operationalized cannot be tested, and this framework is no exception. We therefore define assignment on three measured properties rather than on any single parameter: deformation under a defined load; barrier function, recorded as the frequency and duration of reporter leakage during loading and the completeness of resealing after unloading; and recovery, the operational form of the remodeling capacity introduced in Section 6.1, recorded as restoration of nuclear volume and of repair-factor distribution within a defined post-unload interval. Only barrier function and recovery carry assignment. Deformation is measured but not assigned, because a strain value cannot be separated from hydration, fixation and cell-cycle phase within a single preparation (Section 3.1), and the same value is compatible with either failure mode; its rank nevertheless indicates which failure mode is more likely, high compliance predisposing to leakage and low compliance to restricted recovery (Table 1). State 1 is barrier failure, defined as reporter leakage during loading or incomplete resealing after unloading, and therefore includes both transient rupture with complete resealing and persistent barrier failure; rupture frequency, duration and resealing kinetics should be recorded as modifiers within the state, because they carry different consequences for repair-factor loss even though they do not change assignment. State 2 and State 3 both show no leakage and complete resealing, and are separated by recovery alone.

Three features of this scheme deserve emphasis. Assignment is comparative rather than absolute, because thresholds derived in one cell type do not transfer; each experiment should include a reference population and report the rank ordering of strains and leakage frequencies within that experiment. Mixed or unassignable profiles are also expected rather than exceptional. Barrier status may be indeterminate when leakage is intermittent or close to the detection limit, and a design without an unloading protocol cannot separate State 2 from State 3, so the framework requires that such profiles be retained and reported rather than forced into a single category. Reproducibility should be quantified, for example, by inter-observer agreement on blinded images or by test–retest reliability across independent cultures.

Discriminating experiments follow directly from the axis definitions. Barrier status separates State 1 from State 2 and State 3: reporter leakage during loading, or incomplete resealing after unloading, assigns State 1, whereas State 2 and State 3 show neither. High strain is the expected correlate in State 1 and low strain the expected correlate in State 2, but strain is not used for assignment. Among cells with an intact barrier and complete resealing, a recovery protocol separates State 2 from State 3, since only State 3 predicts restoration of nuclear volume and factor distribution after unloading. Repeated loading serves as an independent confirmatory test rather than a further defining criterion: within cells already assigned to State 1, successive cycles should produce cumulative leakage and progressively less complete reversal, whereas cells assigned to State 3 should return toward baseline after each cycle; the deformation trajectory reported by this test remains a correlate and does not alter assignment.

The claims that these experiments would test differ in evidential status, and the two scales used in this review should be kept apart. Figure 3A depicts the complete design, in which a controlled mechanical perturbation, a defined nuclear phenotype, irradiation, target engagement, damage processing, repair fidelity, and clonogenic survival are all measured in one system, and no study reviewed here attains it. Level I, the level used in Table 2, is deliberately less demanding: it requires controlled mechanical manipulation, irradiation, and a damage-processing endpoint in the same system, and only a minority of studies meet even this. Level II evidence covers mechanisms supported in isolation, such as lamin-dependent changes in 53BP1 availability, force-dependent YAP entry and rupture-associated repair-factor loss, together with mechanical associations with a radiation outcome in which the intervening step was not measured; it supports individual links but not the composite claim. Level III statements, such as those about patient stratification and therapeutic benefit, are hypotheses at a further remove and should be described as such.

### 6.7. Boundaries and Prospective Tests

The framework describes nuclear properties measured under defined conditions, not fixed cell identities. Assignment rules, including any threshold used to implement them, should be fixed before radiation outcomes are assessed and reported alongside those outcomes, so that the state of a cell cannot be read back from how it responded. All three properties are therefore measured, even though two of them carry assignment.

Testing should combine the nuclear measurements with cell cycle, genotype, oxygenation, radiation conditions, repair-factor dynamics, repair products or chromosome abnormalities, and clonogenic survival (Section 7.1.2). The two assignment axes should not be collapsed into a single summary score before the radiation endpoints are read, because a score discards exactly the discordance that mixed profiles express. A nuclear profile sampled once cannot establish that a state is a property of the cells rather than a response to the assay; this limitation and the design that addresses it are treated in Section 7.2.

Reproducibility and validation in independent tumor models and cell-cycle contexts are required before broader application. If the states add no predictive information, cannot be assigned consistently, or depend on thresholds chosen after the outcomes are known, their value will be limited; the key test, developed in Section 7.2, is whether combinations of nuclear properties explain radiation responses better than simpler measures, not whether the third state always produces the highest survival.

## 7. Translational Validation and Future Directions

The three-state model remains a hypothesis-generating framework. It does not yet define validated cell classes, treatment-selection rules, or a general relationship between nuclear mechanics and radiosensitivity. Evidence for target availability, drug development, prognosis, or treatment feasibility should therefore be distinguished from evidence that a nuclear-mechanics-guided intervention improves radiotherapy.

Future work should address three questions: whether mechanical input causes measurable changes in nuclear state and radiation response; whether distinct mechanical variables can be separated experimentally; and whether mechanical–nuclear–repair phenotypes can support patient stratification or treatment. Table 2 records the level assigned to each of the review’s principal claims, using the levels defined in Section 1.4 and operationalized in Section 6.6; Figure 3 shows the matched design that a stricter test of the same claim would require. Each of the following subsections specifies what an affirmative answer would require, rather than assuming that it has already been obtained.

### 7.1. Testing the Causal Chain

#### 7.1.1. Defining Nuclear Mechanical States

Assignment requires barrier status, namely leakage during loading and completeness of resealing after unloading, and recovery of nuclear volume and repair-factor distribution (Section 6.6). The correlates listed there, including load-bearing capacity, deformation and nuclear strain, pore dilation, chromatin remodeling, repair-factor recruitment and damage-site movement, should still be reported, because they can dissociate from barrier status in the same nucleus and indicate which failure mode is likely. Because recovery is itself two-part, a nucleus that restores volume while leaving factor distribution unresolved should be reported as such rather than assigned by default to State 3, and each component should be reported as a separate value.

Molecular tension sensors report loads in selected lamina- or LINC-associated structures, but they do not directly measure whole-nucleus stiffness [202,203,204]. Their signals should therefore be compared with AFM, micropipette aspiration, controlled deformation, or related measurements, and sensor output is a correlate of load transmission rather than a criterion for any state. Nuclear morphology alone cannot reconstruct rupture history or recovery. Studies should report loading geometry, magnitude, duration, recovery interval, cell-cycle phase, and substage where the marker permits, and genotype.

#### 7.1.2. Linking Nuclear State to DNA Damage Processing

Matched experiments should manipulate mechanical conditions and radiation exposure in the same model. Time-resolved measurements should separate initial damage from subsequent processing. Relevant endpoints include DSB recognition, repair-factor recruitment, damage-site movement, pathway use, joining fidelity, residual damage, chromosome abnormalities, and clonogenic survival.

The three states predict different damage-processing phenotypes, summarized in Table 1. Barrier failure (state 1) should increase initial damage and delay re-accumulation of repair factors, with clonogenic outcome depending on recovery capacity. An intact barrier with incomplete recovery (state 2) should reduce damage-site mobility and shift pathway balance, without foci kinetics predicting survival. Complete recovery (state 3) should accelerate damage resolution while preserving fidelity, with the survival effect depending on initial damage and transport rate. These properties should be measured separately rather than inferred from survival.

Different break types may also require different spatial responses. ARP2/3 supports clustering of HR-permissive breaks in mammalian S/G2 cells, while nuclear F-actin and myosin contribute to the relocation of heterochromatic breaks in Drosophila and mouse cells [156,157]. These findings should not be generalized to all radiation-induced DSBs, and the pathway question in Section 4.5 requires a design in which pathway use is measured as a ratio at a defined cell-cycle stage rather than inferred from a single reporter.

#### 7.1.3. Establishing Target Involvement

Causal inference requires perturbing the proposed pathways. Relevant approaches include genetic alteration or depletion of lamin, LINC, membrane-repair, or chromatin-regulatory components; pharmacological inhibition; genetic phenocopy; target-engagement assays; and rescue experiments. Separation-of-function alleles that alter load bearing without altering protein abundance are particularly valuable, because they address the principal confounder in this literature.

Rescue experiments are important for compounds with multiple substrates or off-target effects. Target engagement should be linked to nuclear behavior, DNA-damage processing, and clonogenic survival; a change in nuclear morphology or short-term viability alone does not show that the proposed mechanism mediates the radiation response.

### 7.2. Separating Mechanical Variables

Three-dimensional models should distinguish elasticity, viscoelastic relaxation, ligand presentation, pore structure, transport, metabolism, and oxygenation where possible [18,23,48,49,50]. These variables can change together in matrix systems but may have different effects on mechanosensing, cytoskeletal signaling, nuclear loading, and repair. Fluid shear and interstitial flow should be added to this list, since perfusion changes alter them together with oxygenation (Section 2.2). Reporting should name both the variable that was manipulated and the variable that was measured, because a change in matrix modulus is not a change in the load transmitted to the nucleus.

Studies should also record cell-cycle distribution, genotype, oxygen level, radiation quality, dose, and observation time. Matrix modulus should not be treated as a proxy for nuclear stiffness, and neither should be treated as a proxy for nuclear hydration. Lamin abundance, molecular tension, nuclear deformation, and chromatin compliance are separate measurements. Progerin models show that whole-nucleus or scaffold stiffening can coexist with greater chromatin compliance [201], which is the clearest available demonstration that these variables can diverge. Because barrier status and recovery may themselves vary with cell-cycle phase, assignments should be reported per phase wherever the marker permits rather than pooled across an asynchronous population.

As introduced in Section 6.7, the three-state model should be compared with continuous-variable models and with simpler measures based on matrix stiffness or on individual nuclear properties, and the comparison should specify in advance which metric would count as added information, for example, incremental discrimination over a model already containing matrix modulus and cell-cycle distribution. That comparison should be reported even when it favors the simpler model. Repeated measurements should determine whether a nuclear profile changes after loading, irradiation, or recovery, because a single time point cannot separate a stable state from a transient response. Where a profile does change, the time course rather than the endpoint should be reported, and the post-unload recovery interval used for assignment should be stated explicitly as part of the state definition.

### 7.3. Clinical Translation

#### 7.3.1. Candidate Biomarkers and Imaging

Candidate measurements include lamin A/C abundance and organization, nuclear morphology, YAP nuclear-to-cytoplasmic distribution, and local ECM properties. Lamina- and nesprin-associated sensors can report particular molecular loads [202,203,204], but molecular tension is not whole-nucleus stiffness, and static morphology does not reconstruct rupture history. Table 3 summarizes the information and limitations of these features. The included evidence does not establish any as an independently validated predictor of radiotherapy benefit.

Candidate phenotypes are not validated biomarkers. Prognostic associations and prediction of differential treatment benefit require different evidence. Prognostic biomarkers describe outcome risk, whereas predictive biomarkers concern differential benefit between treatment strategies [206,207,208]. An association in a single cohort receiving the same radiotherapy regimen cannot independently estimate a treatment interaction. Candidate assays require prespecified sampling, analysis, and thresholds, with control of stage, molecular subtype, oxygenation, proliferation, and concurrent treatment. Independent validation should assess calibration, discrimination, and added value beyond existing indicators—criteria that, to our knowledge, none of the candidate nuclear phenotypes has yet met.

Imaging provides a preclinical feasibility example. Mislati et al. linked shear-wave elastographic features to response stratification in a mouse rectal cancer model receiving short-course irradiation [205]. This was an animal study, not a clinical patient cohort, and the measurement does not directly represent nuclear stiffness. Registering local material measurements with pathology, oxygenation, and DDR could help develop multidimensional candidate phenotypes, whose clinical uses require dedicated validation rather than extrapolation from the preclinical setting.

#### 7.3.2. Candidate Therapeutic Strategies

Candidate strategies can be grouped by the level at which they intervene: nuclear structure and chromatin, cytoskeletal or adhesion-associated signaling, and the physical tumor microenvironment. These approaches differ in their mechanistic rationale and in the strength of evidence linking them to radiation outcomes. Target engagement, radiotherapy-specific efficacy, and regulatory or development status are distinct evidence levels and are summarized separately in Table 4. Drug-development status rests on registry entries, prescribing information, and manufacturer releases searched by drug name and trial identifier, all checked on 7 October 2026; clinical activity against a target should not be taken as evidence of benefit when combined with radiotherapy or of selection by nuclear mechanical state.

Interventions targeting nuclear structure or chromatin. These strategies have a direct rationale for altering nuclear material properties or organization, but the evidence does not yet establish that such changes mediate radiotherapy response. Lonafarnib inhibits farnesyltransferase, which participates in prelamin A processing; the enzyme has multiple substrates, so tumor lamin abundance alone cannot predict response [209], and glioma models showed preclinical activity with temozolomide and irradiation whereas NSCLC experiments did not [210,211]. Trichostatin A and valproic acid induced chromatin decompaction and reduced nuclear mechanical responses at small deformations [106], but these findings do not establish that nuclear mechanics mediates the effect of vorinostat combined with irradiation; in the PRAVO phase 1 study, vorinostat with pelvic palliative radiotherapy provided evidence on tolerability and histone acetylation [212] without establishing randomized efficacy or a nuclear-mechanical mechanism. Direct targeting of nesprins, lamins, LEM-domain proteins, ESCRT components or the pore-dilation axis discussed in Section 3.3 remains preclinical [113].

Interventions targeting cytoskeletal and adhesion-associated signaling act upstream of the nucleus, so their effects may vary with cell type and tissue context. ROCK inhibition is one example: the radioprotective effect of fasudil in normal fibroblasts highlights the need to assess tumor control and normal-tissue injury together [75]. Dasatinib increased radiosensitivity in preclinical models, in part by interfering with nuclear localization of epidermal growth factor receptor and blocking DNA repair [213]; however, adding dasatinib to chemoradiotherapy in the phase 2 N0877 glioblastoma trial did not improve overall survival [214]. That result does not rule out a biomarker-selected benefit, but Src inhibition should not be presented as a validated radiosensitizing strategy.

YAP/TEAD targeting belongs to the same signaling-level group. Verteporfin has photodynamic and YAP-independent effects that limit its value as a selective probe [88,215]. The phase 1/2 VT3989 study reported early activity of TEAD targeting in solid tumors, particularly mesothelioma [216], and NCT04665206 was listed as recruiting as of 7 October 2026 [217]; neither establishes radiotherapy benefit selected by nuclear mechanics. Ikena discontinued IK-930 development on 28 May 2024 [218], which records the end of one program rather than failure of the target class.

Interventions targeting the physical tumor microenvironment alter the mechanical or transport conditions that shape cellular loading rather than acting on nuclear components. In ovarian cancer models, losartan reduced collagen and hyaluronan deposition and solid stress while improving perfusion and drug delivery [219], and pegylated hyaluronidase was developed on the same rationale in pancreatic cancer [220]. Because decompression also changes oxygenation and the mechanical stimulus, a radiotherapy effect in such models cannot be attributed to either mechanism alone. Clinical results reinforce the caution: pegvorhyaluronidase alfa did not meet its primary survival endpoint in a phase 3 trial in hyaluronan-high metastatic pancreatic cancer [221], so a plausible mechanical rationale does not by itself predict benefit.

Physical interventions, including low-intensity ultrasound and controlled mechanical loading, may alter perfusion, cellular forces or chromatin accessibility. Ultrasound has been proposed to sensitize tumors through cavitation-related effects, improved perfusion and direct mechanical perturbation, and has reached early clinical testing [222,223]; stretch alters chromatin accessibility and has shown protection against UV-induced or mechanically induced DNA damage in model systems [144,145], but the effect is bidirectional and dose-dependent, and no protocol has established a therapeutic window between tumor and normal tissue.

Taken together, these strategies remain candidates rather than validated radiotherapy interventions. Their evaluation should separate target engagement from radiation-specific benefit and should measure mechanical phenotypes alongside tumor and normal-tissue outcomes. A negative result in an unselected population would not distinguish an inactive mechanism from an unidentified subgroup.

**Table 4:** Mechanistic rationale, radiation evidence, and development status of candidate interventions.

Target Level	Intervention	Actual Evidence and Mechanistic Limits	Regulatory or Development Record
**Nuclear structure and chromatin**	Lonafarnib	Farnesyltransferase inhibition affects multiple substrates. Preclinical glioma activity with temozolomide and irradiation contrasts with absent comparable synergy in NSCLC experiments. Nuclear mechanical mediation is unestablished [210,211].	ZOKINVY is labeled for specified progeria/processing-deficient progeroid laminopathies, not cancer radiosensitization [209].
	Histone deacetylase (HDAC) inhibition/Vorinostat	TSA/VPA decompaction provides nuclear material evidence, not mediation of vorinostat–radiotherapy effects [106]. PRAVO phase 1 supports combination tolerability and pharmacodynamic evaluation, not comparative efficacy [212].	ZOLINZA is labeled for specified cutaneous manifestations of cutaneous T-cell lymphoma after prior treatment, not mechanics-guided radiotherapy [224].
**Cytoskeletal and adhesion-associated signaling**	Fasudil/ROCK	Tumor RhoC–ROCK2 pathway evidence is distinct from fasudil-associated radioprotection in normal human fibroblasts [73,75].	The Japanese ERIL label concerns postoperative subarachnoid-hemorrhage vasospasm and associated ischemic symptoms, not cancer radiotherapy [225].
	Dasatinib/Src family kinase	Preclinical radiosensitization through interference with nuclear epidermal growth factor receptor localization and DNA repair [213]. Randomized phase 2 N0877 found no overall survival benefit when dasatinib was added to chemoradiotherapy in glioblastoma [214].	Approved for specified haematological indications only. Radiotherapy combination tested and negative for overall survival in glioblastoma; biomarker-selected evaluation remains open.
	Verteporfin	YAP-independent effects limit use as a selective mechanistic tool; illumination conditions also matter [88].	VISUDYNE is an ophthalmic photodynamic therapy drug. Its indication does not establish YAP-targeted radiotherapy benefit [215].
	VT3989/IK-930	VT3989 provides early clinical activity for TEAD targeting in solid tumors, rather than validation of mechanics-stratified radiotherapy [216].	NCT04665206 was recruiting [217]. Ikena announced discontinuation of IK-930 development on 28 May 2024 [218].
**Physical tumor microenvironment**	Losartan/ECM normalization	Angiotensin receptor blockade reduces collagen and hyaluronan deposition, lowers solid stress, and improves perfusion and drug delivery in ovarian cancer models [219]. Removal of the mechanical stimulus and improvement of oxygenation occur together, so a radiotherapy benefit cannot be attributed to either alone.	Investigational. No regulatory approval for cancer radiotherapy; the perfusion rationale is preclinical and no randomized radiotherapy trial has tested mechanics-stratified use.
	Pegvorhyaluronidase alfa (pegylated hyaluronidase)	Degrades hyaluronan on the same decompression rationale as matrix normalization [220]. Because decompression alters oxygenation and the mechanical stimulus simultaneously, any radiotherapy effect in these models is mechanistically ambiguous.	Phase 3 trial in hyaluronan-high metastatic pancreatic cancer did not meet its primary survival endpoint [221]. A mechanically plausible rationale did not by itself predict clinical benefit.
	Low-intensity ultrasound	Proposed to sensitize tumors through cavitation-related effects, improved perfusion, and direct mechanical perturbation; these mechanisms are not separable in the reported designs [222,223].	Early clinical testing in several settings; radiotherapy-specific benefit and a tumor–normal-tissue therapeutic window remain unestablished.

Status checked on 7 October 2026. Approval, targetability, combination feasibility, and radiotherapy benefit are distinct conclusions; jurisdictions and specific labels are not interchangeable. Abbreviations: NSCLC, non-small-cell lung cancer; TSA, trichostatin A; VPA, valproic acid; ROCK, Rho-associated kinase; YAP, Yes-associated protein; TEAD, TEA domain transcription factor; ECM, extracellular matrix.

#### 7.3.3. Patient Stratification and Clinical Development Pathway

Clinical development can begin with radiotherapy cohorts using prespecified assays and analyses, followed by independent evaluation of reproducibility and incremental information. Prediction of treatment benefit additionally requires comparable treatment strategies and interaction estimates [206,207,208].

A candidate stratification profile could combine a tissue or matrix property, a nuclear support or barrier feature, a remodeling or repair-factor feature, and a DNA-damage or repair outcome. This composite should be compared with simpler clinical, molecular, and imaging models, and its performance may vary with tumor type, genotype, oxygenation, radiation quality, dose, and fractionation.

Mechanistic window studies and early combination trials should follow only after target engagement, preclinical tumor effects, and acceptable normal-tissue risk have been demonstrated. Studies in immunocompetent animals should measure tumor control, host immunity, and acute and late normal-tissue injury. Photon, proton depth or LET, and high-LET particle responses require separate evaluation rather than extrapolation from a single photon model [133].

The framework will have limited translational value if candidate phenotypes do not improve prediction, interventions do not alter the proposed target or repair process, or tumor sensitization is offset by comparable or greater normal-tissue injury. These criteria direct nuclear mechanotransduction research toward a therapeutic window rather than an isolated positive molecular result, and they distinguish a mechanistic hypothesis from a clinically useful treatment strategy. At present, no candidate phenotype in Table 3 and no intervention in Table 4 has satisfied them, and the framework should be described accordingly in grant applications and clinical proposals.

## 8. Conclusions

Current evidence supports a context-dependent role for the tumor physical environment in radiation response. Through the cytoskeleton–nucleus interface, mechanical conditions may influence nuclear structure and nuclear envelope barrier integrity, chromatin state, damage signaling, and selected DNA repair processes. These effects vary with cellular state, genetic background, material properties, radiation conditions, and experimental endpoints.

The available evidence does not show that any single mechanical parameter universally or unidirectionally determines HR/NHEJ utilization or radioresistance. Damage signaling, repair-factor recruitment, pathway choice, repair fidelity, clonogenic survival, and immune activation represent distinct biological levels. The three-state model of nuclear mechanical homeostasis, comprising insufficient structural support, maladaptive structural constraint, and coordinated integrity and plasticity, therefore provides a provisional and testable framework for organizing these observations, rather than an established explanation of radiation response; its operational criteria are intended to make it falsifiable rather than merely descriptive.

Future studies should measure mechanical state, damage processing, repair-pathway utilization, and clonogenic survival in the same tumor model under matched irradiation conditions. Genetic perturbation, targeted intervention, and rescue experiments will be needed to establish causality, and cell-cycle-resolved phenotyping will be needed to avoid attributing cycle redistribution to nuclear mechanics. Independent validation should then determine whether nuclear mechanical phenotypes can support radiotherapy-specific stratification or intervention while preserving a clinically acceptable tumor–normal-tissue therapeutic window. Physical and pharmacological mechanotherapies, including matrix-normalizing agents, Src inhibitors, and ultrasound-based approaches, are currently the most informative test of the framework’s translational relevance, and the mixed clinical record indicates that they should be evaluated with mechanistic readouts rather than as empirical radiosensitizers.

## Figures and Tables

**Figure 1:**
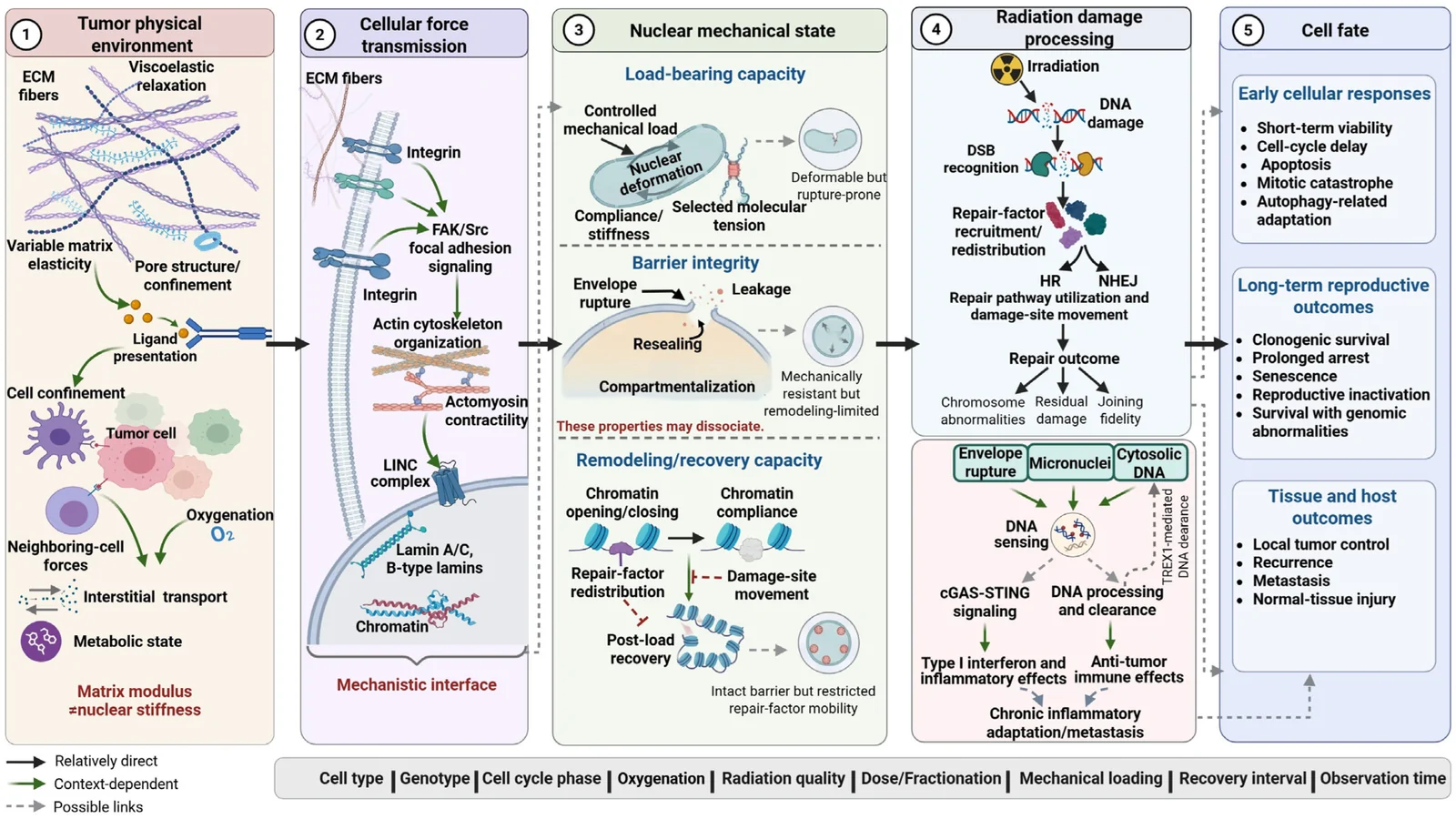
Conceptual overview of nuclear mechanotransduction in cancer radiation response. Matrix mechanics, confinement, ligand presentation, and neighboring-cell forces influence cell adhesion and loading within the tumor physical environment (1), alongside oxygenation, transport, and metabolic conditions. At the cellular interface (2), integrin-mediated adhesion is linked to FAK/Src focal-adhesion signaling, which participates in regulating actin organization. Actomyosin-generated forces reach the lamin–chromatin interface through the LINC complex, connecting extracellular mechanical conditions to nuclear state. The nuclear response to loading (3) encompasses load-bearing capacity, envelope barrier integrity, and remodeling/recovery capacity, which may dissociate, as illustrated by the nuclear sketches. Neither matrix modulus nor a single nuclear stiffness measurement fully captures this state. These nuclear properties may influence post-irradiation damage processing (4), including DNA damage recognition, repair-factor recruitment and redistribution, damage-site movement, and utilization of repair pathways such as HR and NHEJ, with outcomes encompassing residual damage, chromosome abnormalities, and joining fidelity. Disruption of nuclear or micronuclear barriers may also expose DNA to sensing systems, providing another connection between nuclear state and inflammatory or immune effects. This branch is conditioned by pathway activation and DNA processing/clearance, including TREX1-mediated clearance, which can reduce the DNA available for sensing; DNA exposure or cGAS recruitment alone does not establish downstream activation. These damage-processing and immune processes are linked to early cellular responses, long-term reproductive outcomes, and tissue/host outcomes (5), interpreted according to the cellular, irradiation, loading, recovery, and observation conditions shown along the bottom. Black solid arrows indicate relatively direct local relationships, green arrows indicate context-dependent effects, and gray dashed arrows indicate possible links. Red dashed connectors depict schematic functional relationships within the nuclear remodeling/recovery module. The framework integrates evidence across models without establishing a complete causal sequence in a single system or a universal direction of radiosensitivity changes. Gray arrows within the nuclear-state panel schematically relate the illustrated properties to example phenotypes. Abbreviations: ECM, extracellular matrix; FAK, focal adhesion kinase; LINC, linker of nucleoskeleton and cytoskeleton; DSB, DNA double-strand break; HR, homologous recombination; NHEJ, non-homologous end joining; cGAS, cyclic GMP–AMP synthase; STING, stimulator of interferon genes; TREX1, three-prime repair exonuclease 1.

**Figure 2:**
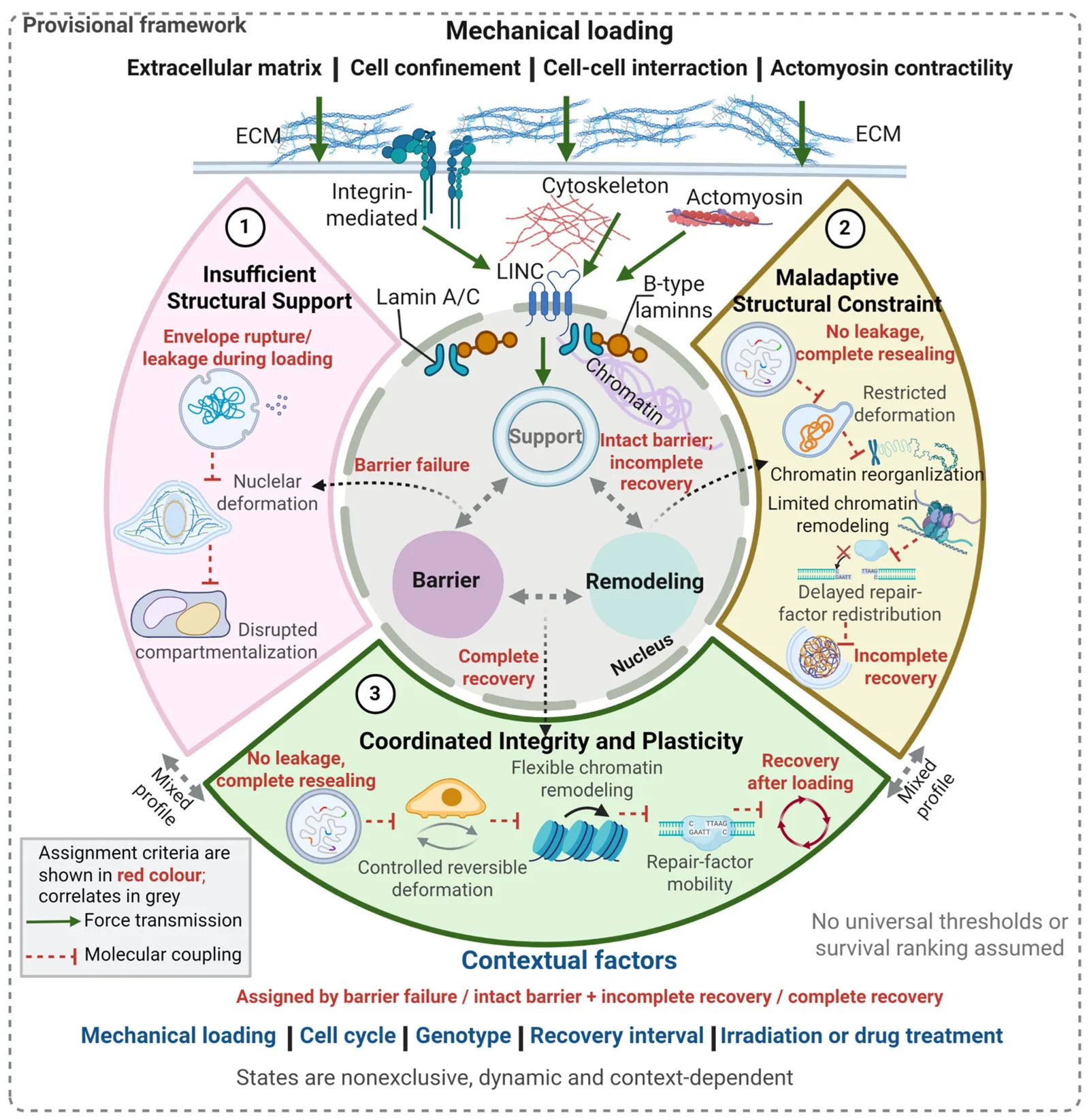
A provisional three-state framework of nuclear mechanical homeostasis. Mechanical inputs from the extracellular matrix, cell confinement, cell–cell interactions and actomyosin contractility reach the nuclear interface through adhesion, the cytoskeleton and the LINC complex (green arrows); red dashed T-ended connectors denote molecular coupling. The central triangle relates three measured properties: structural support, compartmental barrier integrity and remodeling, whose operational form is recovery after unloading. Red marks the assignment criteria and grey the correlates, so barrier status and recovery assign states while support and deformation do not (Section 6.6). State 1 (insufficient structural support) is entered by barrier failure, shown as envelope rupture or leakage during loading, or by incomplete resealing after unloading; state 2 (maladaptive structural constraint) by an intact barrier with incomplete recovery; and state 3 (coordinated integrity and plasticity) by complete resealing with complete recovery. Grey arrows between panels mark mixed profiles, which are reported rather than resolved; states are nonexclusive, and no universal thresholds or survival ranking are assumed. Abbreviations: ECM, extracellular matrix; LINC, linker of nucleoskeleton and cytoskeleton.

**Figure 3:**
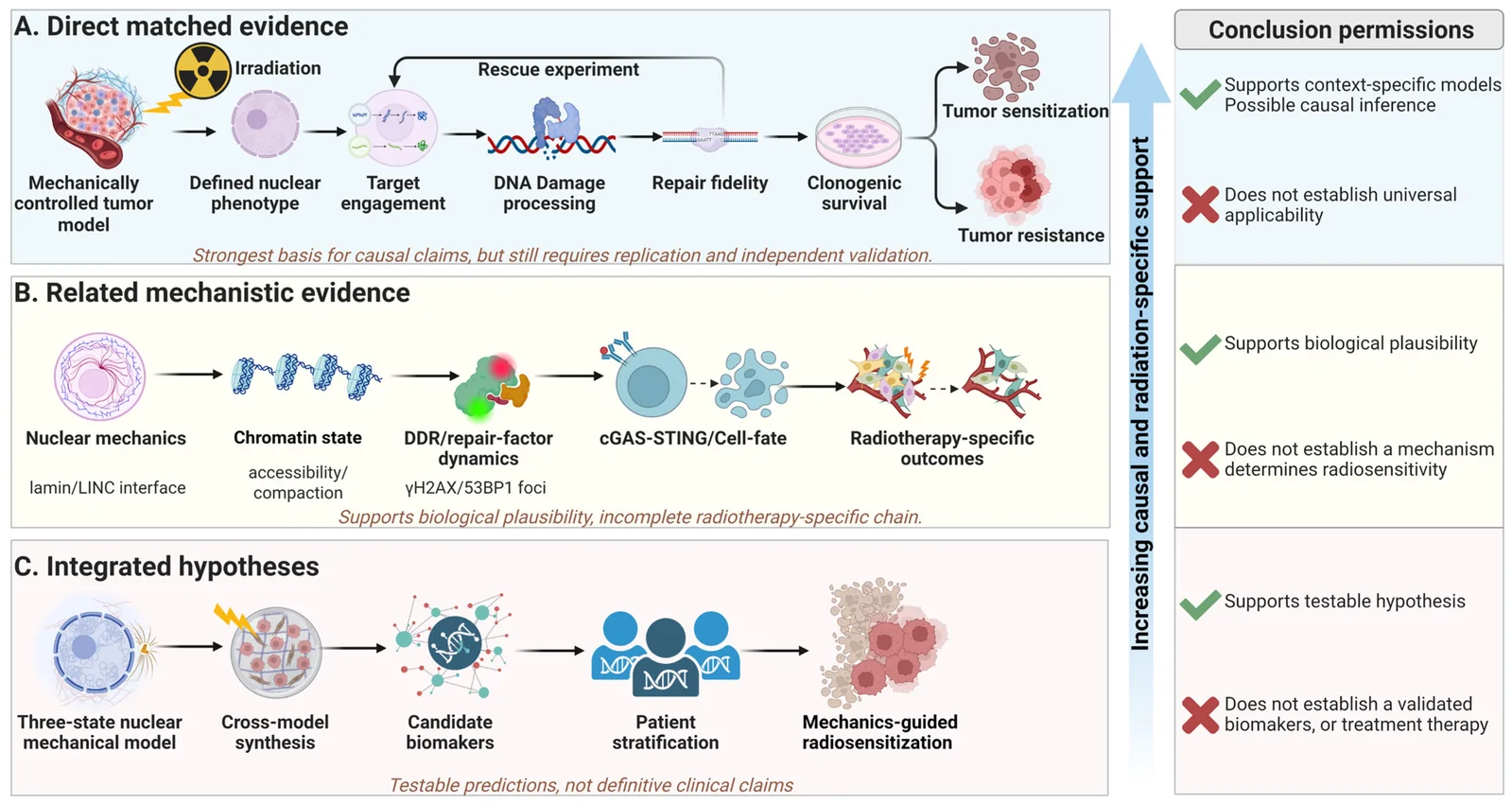
Evidence hierarchy and translational distance in studies of nuclear mechanotransduction and radiation response. Study designs are organized into three categories according to the extent to which mechanical state, irradiation, molecular intermediates, and long-term outcomes are experimentally matched. (**A**) Direct matched evidence combines controlled mechanical perturbation, defined nuclear phenotyping, irradiation, target engagement, DNA-damage processing, repair fidelity, and clonogenic survival, providing the strongest basis for context-specific causal inference while still requiring replication. (**B**) Related mechanistic evidence links nuclear mechanics with chromatin organization, DNA-damage response and repair-factor dynamics, cGAS–STING signaling, or cell fate, but does not by itself establish a radiation-specific causal chain or determine radiosensitivity. (**C**) Integrated hypotheses connect the provisional three-state nuclear model with candidate biomarkers, patient stratification, or mechanics-guided radiosensitization; these relationships generate testable predictions but remain unvalidated. The three categories (**A**–**C**) classify study designs and are not the claim levels used in Table 2; (**A**) shows the complete design; no study reviewed here attains it. Increasing translational distance therefore permits broader hypotheses but weaker causal conclusions: current evidence supports context-dependent biological plausibility rather than universal applicability, validated biomarkers, or clinical treatment strategies. Green ticks indicate interpretations supported by the corresponding evidence, whereas red crosses indicate conclusions that cannot be established from that evidence category alone. Abbreviations: DDR, DNA damage response; LINC, linker of nucleoskeleton and cytoskeleton; cGAS, cyclic GMP–AMP synthase; STING, stimulator of interferon genes; γH2AX, histone H2AX phosphorylated at Ser139; 53BP1, p53-binding protein 1.

**Table 1:** Operational criteria, discriminating measurements, and predicted radiation phenotypes for the three proposed nuclear mechanical states.

Proposed State	Operational Criterion (Assigned Before Irradiation)	Discriminating Measurement and Predicted Radiation Phenotype
State 1: insufficient structural support	Barrier leakage during loading or incomplete resealing after unloading, with high nuclear strain as the expected correlate. Assignment uses comparative rank order within one experiment, not fixed thresholds.	Strain imaging and leakage reporter measured simultaneously, plus post-unload resealing kinetics. Prediction: greater initial damage from deformation- and rupture-associated breaks, not from increased radiation energy deposition, and delayed re-accumulation of repair factors; clonogenic outcome depends on recovery capacity. Confounders: cell-cycle phase, hydration, fixation.
State 2: maladaptive structural constraint	Intact barrier during loading, with incomplete recovery of nuclear volume or of repair-factor distribution after unloading. Low strain under load is the expected correlate.	Strain measurement combined with photobleaching or photoconversion of one repair factor at damage sites, plus post-unload nuclear volume recovery. Prediction: reduced damage-site mobility and a shift in pathway balance; foci kinetics do not predict survival. Confounders: lamin A-to-B ratio, chromatin compaction, progerin.
State 3: coordinated integrity and plasticity	Intact barrier during loading with complete resealing, and complete recovery of nuclear volume and factor distribution within a predefined interval; the strain rank is not part of the criterion.	Repeated loading–recovery protocol with recovery of reporter distribution. Prediction: faster damage resolution with preserved fidelity; survival effect depends on initial damage and on transport rate. Confounder: nucleolar stress response.
Mixed or unassignable	Barrier status indeterminate, or no leakage detected but recovery not assessed.	All three properties reported separately without forced assignment; inter-observer agreement or test–retest reliability reported. Prediction: exploratory only, and excluded from primary inference.

**Table 2:** Core claims of this review, the level of evidence supporting each, and the studies on which the claim rests.

Core Claim	Level	Key Evidence and Its Limits	Refs.
Matrix stiffness alters radiation response, but the direction differs between models	II	Conflicting directions across tumor models;	[11,12,20,23,24]
β1-integrin and FAK signaling modify post-irradiation survival	II	Receptor and kinase perturbations combined with irradiation, but the load transmitted to the nucleus is not measured	[61,62,63,64,65,66,67]
Persistent RhoA inhibition or activation reduces post-irradiation reproductive survival	II	Both inhibitory and activating RhoA mutants radiosensitize; no nuclear readout, so the mechanism remains unattributed	[72]
Shear stress applied before irradiation radiosensitizes through integrin and FAK	II	Flow-chamber and microfluidic systems rather than irradiated tumors	[38]
Intermediate filaments set nuclear deformation and envelope integrity	II	Keratin, vimentin and glioblastoma models; no radiation endpoint	[92,93,94]
LINC transmits load and separately contributes to the damage response	II	Mechanical and scaffolding functions have not been separated within a single allele	[26,98,110]
Pore dilation changes permeability and nucleocytoplasmic flux under load	II	Reversible and distinct from rupture; radiation relevance untested	[112,113]
Low stiffness suppresses RNF8-dependent ubiquitin signaling and lowers repair reporter activity	I	Manipulation, irradiation, pathway reporters, residual damage and survival in one system; joining fidelity not measured	[13]
Stiffness-driven PFKFB3- and Ku70-associated signaling alters post-irradiation damage processing	I	Hydrogel substrates with γH2AX, comet and growth endpoints; accurate c-NHEJ not demonstrated	[12]
Stiffness-dependent YAP/TEAD signaling sustains post-irradiation growth in glioma stem cells	I	Material manipulation with genetic perturbations and irradiation; no repair-fidelity endpoint	[11]
PIEZO1 activation radiosensitizes melanoma cells and tumors	I	Knockdown, damage and colony endpoints plus tumor growth, although the input is osmotic rather than purely mechanical	[80]
A three-state model of nuclear mechanical homeostasis reconciles the conflicting findings	III	Hypothesis with proposed operational criteria; not yet tested prospectively	Figure 2

Note: Levels are defined in Section 1.4 and operationalized in Section 6.6: I, controlled mechanical manipulation and irradiation combined with a damage-processing endpoint in the same system; II, either a mechanosensitive nuclear or damage-response mechanism or an association between a mechanical variable and a radiation outcome, where a matched damage-processing endpoint was not measured in the same system; III, an integrated hypothesis that remains untested. These levels classify claims and are distinct from the study-design categories (A–C) of Figure 3. Abbreviations: FAK, focal adhesion kinase; LINC, linker of nucleoskeleton and cytoskeleton; RNF8, RING finger protein 8; PFKFB3, 6-phosphofructo-2-kinase/fructose-2,6-bisphosphatase 3; YAP, Yes-associated protein; TEAD, TEA domain transcription factor; PIEZO1, piezo-type mechanosensitive ion channel component 1; γH2AX, histone H2AX phosphorylated at Ser139; c-NHEJ, classical non-homologous end joining.

**Table 3:** Candidate phenotypes related to nuclear mechanotransduction and limits of interpretation.

Candidate Feature	Measurement and Mechanistic Information	Radiotherapy Interpretation and Limitations
Lamin A/C and B-type lamins	Staining, protein abundance, and spatial organization reflect lamina composition and selected repair-factor states [40,41,42,43,71,99,109].	Abundance is not stiffness. Isoforms, processing, and factor sequestration can have different DDR effects. Independent radiotherapy cohorts and treatment-interaction validation are needed.
Nuclear morphology, deformation, and barrier recovery	Pathology or live imaging combined with controlled loading, leakage, and resealing separates static shape from dynamic recovery [29,31,33,117,118,119].	Irregular shape or one rupture does not define sensitivity. Genotype, cell cycle, migration, and sample preparation affect measurements.
YAP nuclear-to-cytoplasmic distribution	Imaging with TEAD-related transcription and sampling time characterizes localization and adaptation [11,27,45,46,81,82,83,84,85,86,87].	Localization alone cannot separate mechanical input, irradiation-induced translocation, and persistent memory. Pharmacological specificity requires separate assessment.
ECM and tissue elastic features	Local material measurements or elastography, with collagen, hyaluronan, and oxygenation; mouse rectal cancer provides a radiation-response imaging example [14,15,16,17,18,19,20,21,22,48,205].	Tissue modulus is not nuclear stiffness. Heterogeneity and perfusion can affect associations; animal stratification does not establish clinical predictive utility.
Lamina/LINC molecular tension	Specific constructs report molecular load and complement whole-nucleus deformation measurements [202,203,204].	Measurements depend on sensor design and calibration. Mechanistic feasibility does not establish a routine patient assay or standalone predictor of radiation benefit.

Abbreviations: DDR, DNA damage response; YAP, Yes-associated protein; TEAD, TEA domain transcription factor; ECM, extracellular matrix; LINC, linker of nucleoskeleton and cytoskeleton.

## Data Availability

No new data were created or analyzed in this study. Data sharing is not applicable to this article.
